# Impacts of Sexual and Reproductive Health and Rights Misinformation in Digital Spaces on Human Rights Protection and Promotion: Scoping Review

**DOI:** 10.2196/83747

**Published:** 2025-12-30

**Authors:** Tina D Purnat, Elisabeth Wilhelm, David Scales, Claire Wardle, Sheri Bastien, Bela Ganatra, Antonella Lavelanet, Gitau Mburu, Tigest Tamrat, Åsa Nihlén

**Affiliations:** 1 Harvard TH Chan School of Public Health Boston, MA United States; 2 Department of Midwifery School of Healthcare and Care Sciences University of West Attica Athens Greece; 3 Weill Cornell Medicine New York, NY United States; 4 Department of Communication Cornell University Ithaca, NY United States; 5 UNDP (United Nations Development Programme)/UNFPA (United Nations Population Fund)/UNICEF (United Nations Children's Fund)/WHO (World Health Organization)/World Bank Special Programme of Research, Development and Research Training in Human Reproduction Department of Sexual, Reproductive, Maternal, Child, Adolescent Health and Ageing World Health Organization Geneva, GE Switzerland

**Keywords:** communication, sexual health, reproductive health, human rights, information science, infodemic, digital spaces, information environment, misinformation, information ecosystem, digital platforms, digital communities

## Abstract

**Background:**

Sexual and reproductive health and rights (SRHR) are foundational to both individual autonomy and global well-being. Misinformation in this domain poses serious risks by undermining evidence-based decision-making, weakening systems of accountability, and perpetuating social injustices.

**Objective:**

This scoping review aimed to map and synthesize evidence on the forms, spread, and impacts of misinformation related to SRHR in digital spaces, with a particular focus on implications for the protection and promotion of human rights.

**Methods:**

We conducted a scoping review of scientific papers and gray literature. It was guided by the JBI (Joanna Briggs Institute) population, exposure, and outcomes framework. The extracted information was documented following the PRISMA-ScR (Preferred Reporting Items for Systematic Reviews and Meta-Analyses extension for Scoping Reviews) checklist. Thematic analysis was carried out and mapped against human rights standards: (1) equality and nondiscrimination; (2) Availability, Accessibility, Acceptability, and Quality; (3) informed decision-making; (4) privacy and confidentiality; (5) participation and inclusion; and (6) accountability.

**Results:**

Of the 254 eligible studies and documents, 133 focused on the information ecosystem, 37 on the individual, 32 on service delivery and health system, 31 on law and policy, and 21 on community levels. SRHR misinformation impacts individuals’ informed SRHR decisions by shaping their beliefs, attitudes, and health-seeking behaviors. It reinforces harmful and discriminatory social norms at community levels and the exclusion of marginalized voices. SRHR misinformation impacts health systems by shaping provider knowledge and practice, disrupting service delivery, and creating barriers to equitable care. It may function as a legal and policy tool to erode SRHR protections. The design of online platforms, digital marketing strategies, and content moderation policies enables misinformation to spread widely while restricting credible SRHR content.

**Conclusions:**

SRHR misinformation in digital spaces is a systemic issue that undermines human rights across multiple levels, highlighting the urgent need for integrated, rights-based approaches to research, policy, and intervention.

## Introduction

Access to accurate, evidence-based information is integral to realizing sexual and reproductive health and rights (SRHR). The ability to make informed decisions across the full spectrum of sexual and reproductive health (SRH)—from the prevention of health-harmful practices to contraception, fertility, pregnancy, and lifelong sexual health and well-being—is fundamental to health, dignity, population resilience, and enables people to thrive into the future [1]. Misinformation [2] in this domain can pose significant risks by undermining evidence-based decision-making, eroding accountability, and perpetuating injustices. Although public health programs have thorough experience and expertise in providing credible, accurate health information to clinicians, patients, and the public, these long-standing approaches have been strained in the past two decades by rapidly changing information ecosystems [3-5]. How people seek and consume health information has changed with the digitization of information exchange and the ubiquity of consumer-centered and device-mediated information consumption. Digital access to health information is particularly critical for adolescents, who rely on online sources for education and support [6].

Misinformation is defined as false, inaccurate, or misleading information shared without intent to deceive [2]. It differs from disinformation, which involves the intentional spread of false or misleading content to profit from, deceive, or manipulate individuals or communities [2]. While these two concepts are distinct in terms of intent, they often operate in overlapping and reinforcing ways within digital environments and leverage the same features of the information ecosystem and vulnerabilities of online users [7]. In practice, the outcomes of both misinformation and disinformation, such as confusion, mistrust, stigmatization, and harm, can be similarly damaging, especially in the context of SRHR. SRHR disinformation is included in this review as a particularly harmful form of misinformation, with the potential to deliberately erode human rights protections and restrict access to evidence-based care.

Both mis- and disinformation spread rapidly in digital spaces, which are online environments where people interact, share content, and consume information. These include social media platforms (eg, Facebook [Meta], X [formerly known as Twitter; X Corp], and Instagram [Instagram from Meta]), websites, forums (eg, Reddit [Reddit, Inc], comment sections under news or blog posts), blogs, messaging apps (eg, WhatsApp [Meta] and Telegram [Telegram Messenger Inc]), and other digital platforms (eg, Alexa [Amazon.com, Inc] and Google Maps [Google Inc]). However, this digital ecosystem is far broader than traditional social media: people also encounter and negotiate SRHR narratives in the digital information environment within gaming communities, livestreaming platforms, pornography sites, dating apps, and short-form video environments such as TikTok or YouTube Shorts (Google LLC). These varied spaces are not only sites of information transmission, but also of identity formation, social bonding, entertainment, and emotional expression, all of which shape how users interpret and act on health-related messages. For many individuals, particularly youth and marginalized communities, these platforms have become primary sources of SRHR knowledge, community, and meaning-making. Yet, they also enable the amplification of inaccurate or deceptive content, often without adequate moderation or accountability. This can distort individuals’ perceptions, influence health-related decisions and behaviors, reinforce harmful norms and stigma, and erode trust in public health guidance and institutions [8,9].

The broader field of misinformation research is rapidly evolving. It is moving beyond static definitions of false content and intentions to spread it to embrace a more dynamic, contextualized understanding of how information and narratives flow through digital spaces and impact social norms and behaviors [3], and learning from specific health contexts such as vaccine demand promotion, science communication, cancer treatment and prevention, and pandemic preparedness and outbreak response [4,10-14]. Scholars and public health leaders increasingly recognize that interventions focused solely on individual knowledge, fact-checking, or improved digital marketing are insufficient in addressing the structural and systemic nature of the problem [3,9,14-17]. Misinformation does not merely reflect gaps in public understanding, but intersects with identity, emotion, prior experience, and the relational dynamics that shape trust and meaning-making [3,8,16,18].

Public health responses to misinformation have often focused on correcting individual behaviors rather than addressing systemic failures in information governance [14,15,17,19]. Public health efforts that continue to emphasize scale through adaptable digital communication and engagement risk replicating limited impact if they do not adapt to the lived realities of how people seek, share, and interpret health information and experience relationships online [15,16,18]. This shift in understanding has catalyzed calls for more equity-centered, interdisciplinary, and reflexive approaches that engage with information ecosystems holistically, recognize user agency and curation, and address power imbalances in how knowledge is produced and accessed [2,3,14-18,20].

The information environment refers to the dynamic set of ecosystems [21] of all the communication channels, platforms, actors, narratives, and interactions that influence how individuals receive, process, and use information. Often, public health functions do not have strong policies or funded programs designed to address information environment challenges or mitigate their harms to health, and many new efforts were stood down after the pandemic due to a lack of resources and integration into routine programming [5,17,19,22].

In the context of SRHR, the information environment includes not only digital platforms but also how it intersects with traditional media, community networks, health systems, legal frameworks, socially built identities, and social norms that shape the flow and interpretation of SRHR information. Additionally, the World Health Organization (WHO) has identified artificial intelligence (AI)–generated misinformation as a growing threat to SRHR [23], and the United Nations (UN) has recognized the role of digital platforms in shaping the SRHR information landscape and has raised concerns about governance and accountability [24]. Companies that operate these platforms have a responsibility to respect human rights; yet, content moderation policies and advertising models often allow misinformation to proliferate [25,26].

This scoping review maps existing literature on how misinformation related to SRHRs circulates in digital spaces, with the aim of identifying dominant themes, gaps in the literature, and implications for human rights protection (reducing harm) and promotion (enabling autonomy). It introduces human rights standards to an analysis in information science and misinformation studies, and is not an interpretation of existing legal frameworks that are used in international law in relation to SRH and health information. At the same time, it adds a socioecological lens to the analysis of SRH misinformation literature, which enables a more comprehensive discussion of influences and impacts at each of the socioecological levels, as well as interactions between them.

This paper is the first in a series of publications that will emerge from the data collected during the review. This paper examines SRHR misinformation through the levels of the socioecological model and aims to inform future research priorities and guide the development of rights-based, evidence-informed interventions in an increasingly complex and rapidly evolving digital information landscape. While this paper focuses on the human rights impacts of SRHR misinformation, future papers from this review will map, describe, and discuss the drivers and amplifiers of SRHR misinformation, and interventions and recommendations.

## Methods

### Study Team

The core study team consisted of five members with expertise in health communication, misinformation studies, community engagement, information science and analytics, medicine, sociology, SRH, gender, and human rights. A project advisory group was convened and consulted in defining the scope, distributing a web survey to networks and experts, reviewing findings, and reporting results. Consultations with SRHR practitioners and policy makers, human rights experts, and digital health and health communication specialists were conducted to identify additional sources of gray literature, validate findings, and provide contextual insights on addressing SRHR misinformation.

### Study Design

We used the JBI (Joanna Briggs Institute) manual for evidence synthesis for the overall process of review and analysis [27]. We followed the PRISMA-ScR (Preferred Reporting Items for Systematic Reviews and Meta-Analyses extension for Scoping Reviews) checklist [28] (Multimedia Appendix 1).

The objective was to organize and summarize available literature and inform future research on the incidence, types, and influence of SRHR misinformation in digital spaces and its effects at the individual, community, health system, law and policy, and information ecosystem levels, from a human rights perspective.

### Identifying Research Questions

The following questions guided the scoping review: (1) What are the common types of SRHR misinformation and narratives in the digital information environment? (2) How is SRHR misinformation amplified in the digital information environment, and why or for what reasons? (3) What kind of effect does SRHR misinformation and narratives in the digital information environment have on individuals, communities, service delivery, laws, and policy?

To effectively answer these questions, we adopted the population, exposure, and outcome framework developed by the JBI [29]. The population, exposure, and outcome criteria were as follows: P (population)—research and gray literature focused on SRHR misinformation in digital information environment affecting individuals, communities, health workers, or policy makers; E (exposure)—misinformation related to SRHR topics such as contraception, fertility, abortion, maternal health, sexual health, and bodily autonomy; and O (outcomes)—impacts on decision-making, health-seeking behavior, health service delivery, community norms, laws, and policies.

The corresponding inclusion and exclusion criteria are summarized in the SRHR misinformation Table 1.

**Table 1:** Summary of inclusion and exclusion criteria for papers in this scoping review focused on misinformation in digital spaces related to sexual and reproductive health and rights.

	Included	Excluded
Paper type	Original research papers, policy analyses, policy reports, and commentaries published in peer-reviewed research journals and gray literature	Preprints, duplicates, theses, or book chapters
Methodology	Can include any methodology or type of research (qualitative, quantitative, and mixed methods)	N/A^a^
Geography	Any country or region	N/A
Language	English	Not in English
Publication date range	January 1, 2019, and November 23, 2024	Outside of January 1, 2019, and November 23, 2024
Full text available?	Yes	No
Related to topics in sexual and reproductive health and rights (eg, contraception, fertility, abortion, maternal health, and bodily autonomy)	Yes	No
Related to misinformation in digital spaces (eg, includes social media, apps, and the internet)	Yes	No
Related to health information–related outcomes? (eg, decision-making, health-seeking behavior, health service delivery, community norms, laws, and policies)	Yes	No

^a^N/A: not applicable.

### Search Strategy

We considered qualitative, quantitative, and mixed-methods studies, commentaries, policy analyses, and gray literature in English. We searched PubMed, Scopus, Web of Science, JStor, and HeinOnline databases with a search strategy presented in Multimedia Appendix 2. In addition to more traditionally used databases in information science (PubMed, Scopus, and Web of Science), additional searches were also performed in JStor and HeinOnline. JStor, as Web of Science, covers humanities and social sciences, but it is more expansive, which was deemed useful to locate more papers for this study. HeinOnline covers legal and law-related materials and was included to search for current discussions of SRHR misinformation in digital spaces, as impacts on law were part of the research question.

Search terms were tested and developed iteratively from narrow to the final broader definitions to intentionally capture the diversity of studies that might otherwise not be included because they used diverse keywords and terms in relation to SRH and misinformation. We included studies and documents published and made available in the databases between January 1, 2019, and November 23, 2024.

Information environment dynamics, related regulatory, platform, and consumer protection policies, user trends, and narratives change quickly and are often first described in gray literature before scientific papers. As we wanted to capture the current discussions and evidence, and the SRHR misinformation impacts are often described in gray literature such as relevant government, UN, and nongovernmental organization reports and newspapers, we also searched Google and Google Scholar. In addition, policy and evidence reports were also suggested by key informants via a web survey on the WHO website that was open for 6 weeks, between November 25, 2024, and January 5, 2025, and advertised through WHO SRHR networks and social media.

### Document Screening and Inclusion

Two investigators searched and screened the documents by titles and abstracts, and then reviewed the full texts of potential documents to include. The screening by titles and abstracts focused on excluding and marking as irrelevant the studies that did not relate to SRHR misinformation and digital spaces, were in a language other than English, or that were books, book chapters, or theses. The subsequent full-text review focused on applying all exclusion and inclusion criteria to the remaining studies. Conflicts were resolved through consensus between the two investigators. Records were managed in the Covidence systematic review software [30]. We excluded any documents not in English, reviews and meta-analyses, and those that did not focus on misinformation in the digital information environment or misinformation in SRHR.

### Data Extraction

A data extraction form was developed to extract data from included studies. The form included fields to capture information about the study or document (authors, title, journal or publisher, and country or region) and document or study characteristics (study type, digital setting, and human rights standards). It also captured misinformation characteristics (topics and types), underlying drivers and attributions, digital channel, impacts of misinformation, affected populations (by social group, identity, or vulnerability), legal and policy implications, interventions and solutions, and additional notes.

### Summarizing and Reporting the Results

We conducted a thematic content analysis using narrative descriptions of the extracted data, and mapped it against categories of human rights standards, namely (1) equality and nondiscrimination, (2) Availability, Accessibility, Acceptability, and Quality (AAAQ), (3) informed decision-making, (4) privacy and confidentiality, (5) participation and inclusion, and (6) accountability. The sources for the human rights standards applied include international and regional human rights treaties, the general comments and recommendations issued by the UN human rights treaty-monitoring bodies, international and regional court decisions, and international and regional consensus documents. The application of these to the area of SRH was made on the basis of WHO guidelines, taking “Ensuring Human Rights in Contraceptive Information and Services: WHO Guidance and Recommendations” (2014) [31] as a key reference and point of departure. We organized the results by levels of the socioecological model: individual, community, service delivery and health system, law and policy, and information ecosystem. The work was performed by two analysts and reviewed by two other reviewers.

### Patient and Public Involvement

This study did not involve patients or the general public. Their input was not sought in the scoping review design, interpretation of results, or drafting or editing this document.

### Ethical Considerations

This study was a scoping review of publicly available literature and documents, and ethical approval was not required.

## Results

### Overview

**Figure 1:**
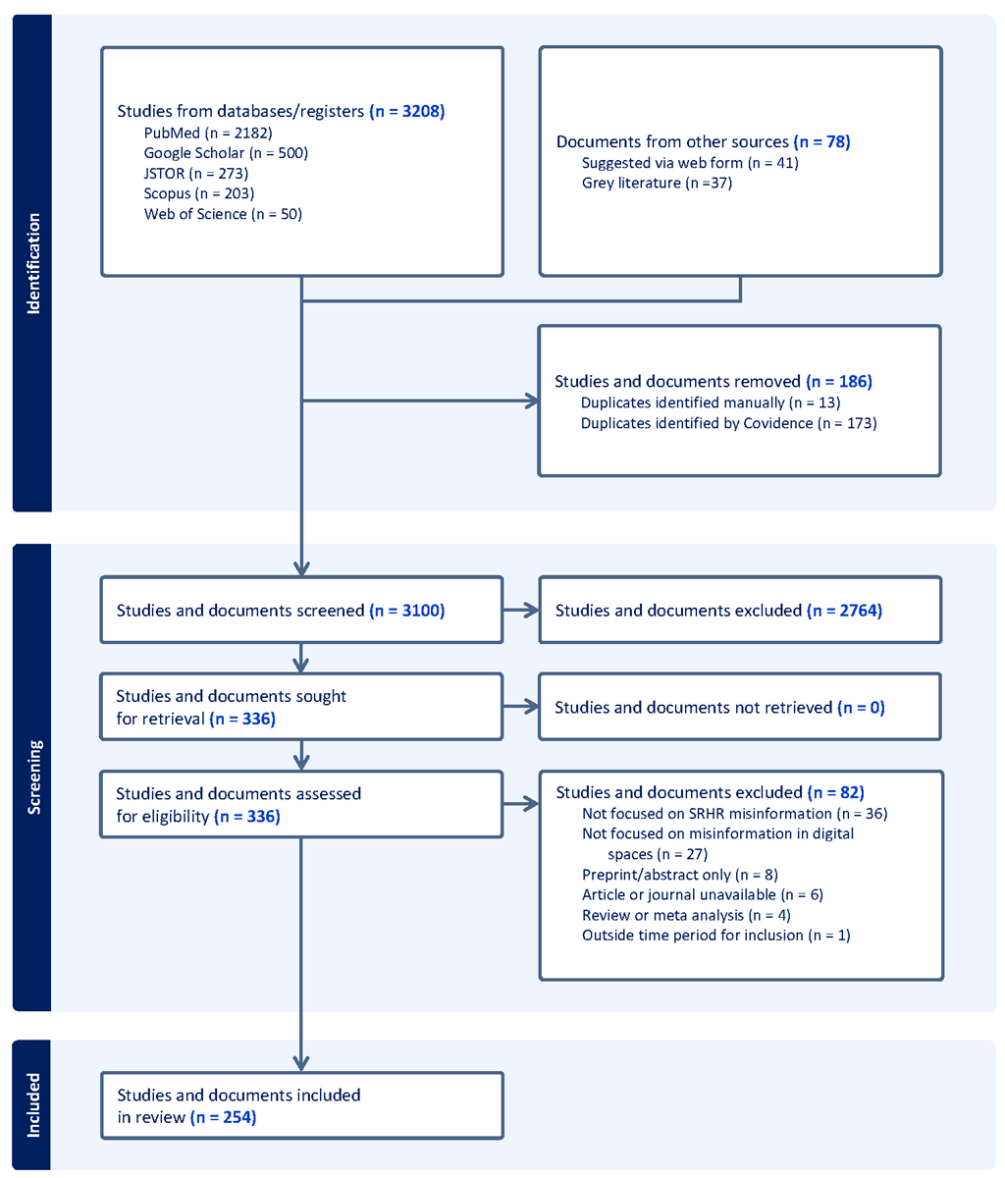
PRISMA-ScR flow diagram. PRISMA-ScR: Preferred Reporting Items for Systematic Reviews and Meta-Analyses extension for Scoping Reviews; SRHR: sexual and reproductive health and rights.

We retrieved 3208 studies from databases and an additional 78 documents from gray literature sources. After removing duplicates, 3100 records remained for screening of titles and abstracts, of which 336 were retrieved for full-text screening. A total of 82 records were excluded for the following reasons: not focused on SRHR misinformation (n=36), not focused on misinformation in digital spaces (n=27), was a preprint or only had an abstract available (n=8), unavailable to retrieve (n=6), review or meta-analysis (n=4), outside of time period for inclusion (n=1). In total, 254 studies and documents were included in the final review. The study selection process is outlined in the PRISMA-ScR flow diagram in Figure 1.

### Description of Included Studies and Documents

#### Characteristics of Studies and Documents

Of the 254 studies and documents (Multimedia Appendix 3 [23,24,32-278]), 77% (n=195) were from scientific journals and 23% (n=59) from gray literature. Detailed figures describing the studies and documents are listed in Multimedia Appendix 4. All analysis was carried out on the full dataset of studies and documents, and referred to as “studies” for the rest of this paper.

Not all studies referred to specific geographies; 43% (n=40) had a global focus, 39% (n=37) focused solely on the US context, and 18% (n=16) focused on other country contexts.

In total, 45 countries or regions were covered in the studies. The 5 countries included in most studies were the United States (n=80), Canada (n=12), Australia (n=9), Brazil (n=7), and the United Kingdom (n=5), followed by studies of the digital environment spanning the European region (n=5). Only 10% (n=26) of studies and documents specified the language of the misinformation that was covered; 12 analyzed English content, followed by Spanish (n=4), French (n=4), Portuguese (n=2), Dutch (n=1), Russian (n=1), Malay (n=1), and Ukrainian (n=1).

Among the included studies, 49% (n=125) are qualitative and focused on the description of digital SRH misinformation in specific populations, specific settings, and on specific SRH topics. Other types of studies were quantitative studies (n=27), news or magazine articles (n=25), commentaries (n=24), mixed-methods studies (n=16), reports (n=15), policy analyses (n=8), legal analyses (n=8), advocacy materials (n=2), webinars or podcasts (n=2), and toolkits or tools (n=2).

More than half (n=149, 57%) of the 254 studies described specific challenges or characteristics of online SRHR misinformation but did not make any recommendations or list strategies for action. A total of 86% (128/149) of those were from scientific journals.

#### Characteristics of SRHR Misinformation

**Figure 2:**
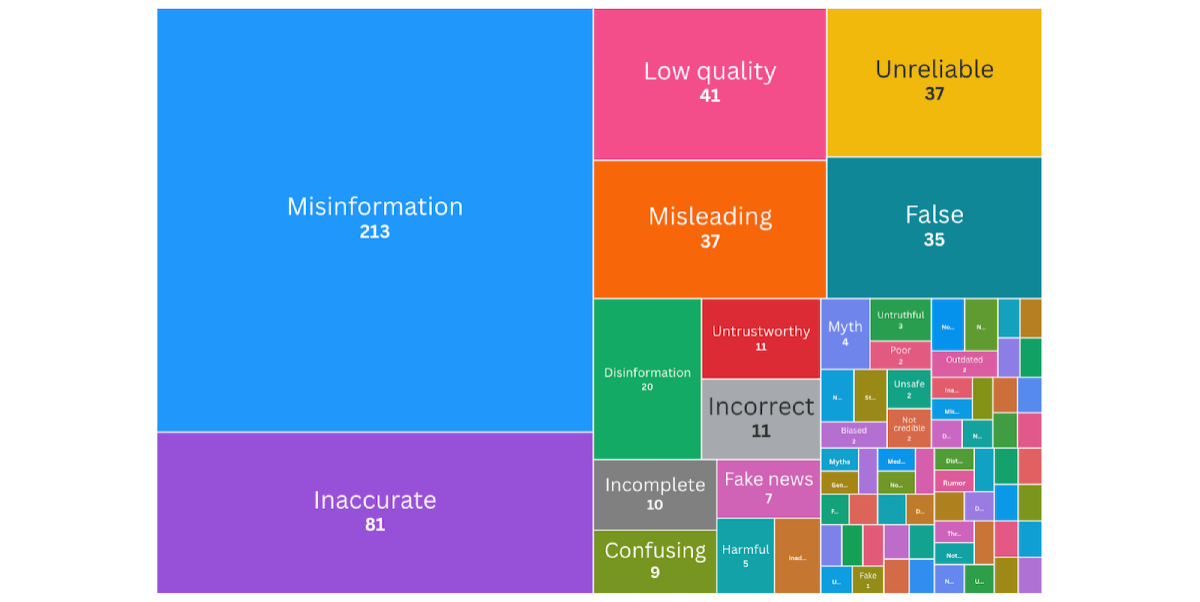
A treemap of terms used to refer to SRH misinformation in studies included in the scoping review. The number next to each term represents the count of the studies that used it when referring to misinformation. SRH: sexual and reproductive health.

For each study, terms that were used to describe misinformation were logged. Across all studies, 75 different terms were used to refer to misinformation, and often multiple terms within the same study. A treemap was generated with the Fourish [279] web tool, where the number of studies (of total 254) that used each term is represented with a proportionally-sized box (Figure 2). Underlying data for the treemap is in Multimedia Appendix 4.

The SRH topics that were covered by the studies were very diverse and are summarized in Multimedia Appendix 4, by topics referred to by authors or communities described in the study. More than 25% (n=72) of all studies discussed SRHR in broad terms, while those focusing on specific topics included maternal and perinatal health (n=36), safe abortion and postabortion care (n=30), fertility, infertility and assisted reproduction (n=20), vaccines (n=19), contraception and family planning (n=17), men’s SRH (n=16), adolescent SRH (n=13), LGBTQ+ (Lesbian, Gay, Bisexual, Transgender, Queer, and more) SRH (n=12), gender-based violence and sexual violence (n=6), sexually transmitted infections, including HIV (n=4), menstrual health and hygiene (n=3), cervical cancer and human papillomavirus (HPV) prevention (n=2), and SRHR in public health emergencies (n=1).

#### Digital Contexts and Settings

Regarding digital information environment or settings covered, the most commonly discussed were digital platforms in general (n=151, 59%) and social media (n=98, 35%), while far fewer discussed search engines (n=8, 3%), advertising and marketing (n=8, 3%), and online forums (n=6, 2%). If specifically mentioned, 23 different platforms were discussed across all studies and documents, most commonly X (formerly known as Twitter; n=32), Facebook (n=28), YouTube (Google LLC; n=26), and TikTok (n=26).

**Figure 3:**
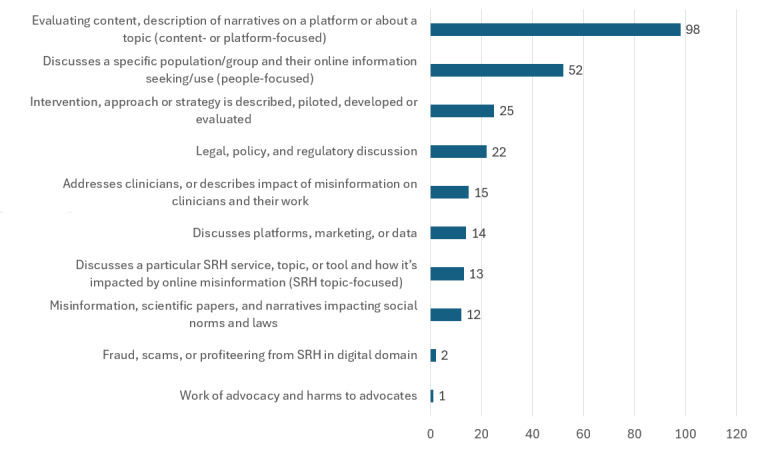
Exploratory categorization of types of misinformation discussion across included studies and documents. SRH: sexual and reproductive health.

An exploratory categorization of the studies and documents by topic discussed in relation to misinformation was developed as a preliminary overview, as existing taxonomies are not integrative and omit aspects that would need further coding in this dataset. This exploratory categorization showed that the most common types of study were about content- or platform-focused issues, how specific groups use digital spaces for SRH, descriptions of interventions and strategies, and legal and policy discussions (Figure 3). The exploratory categorization will be discussed in more detail in future papers.

### Human Rights Impact of SRHR Misinformation by Level of the Socioecological Model

#### Overview

**Figure 4:**
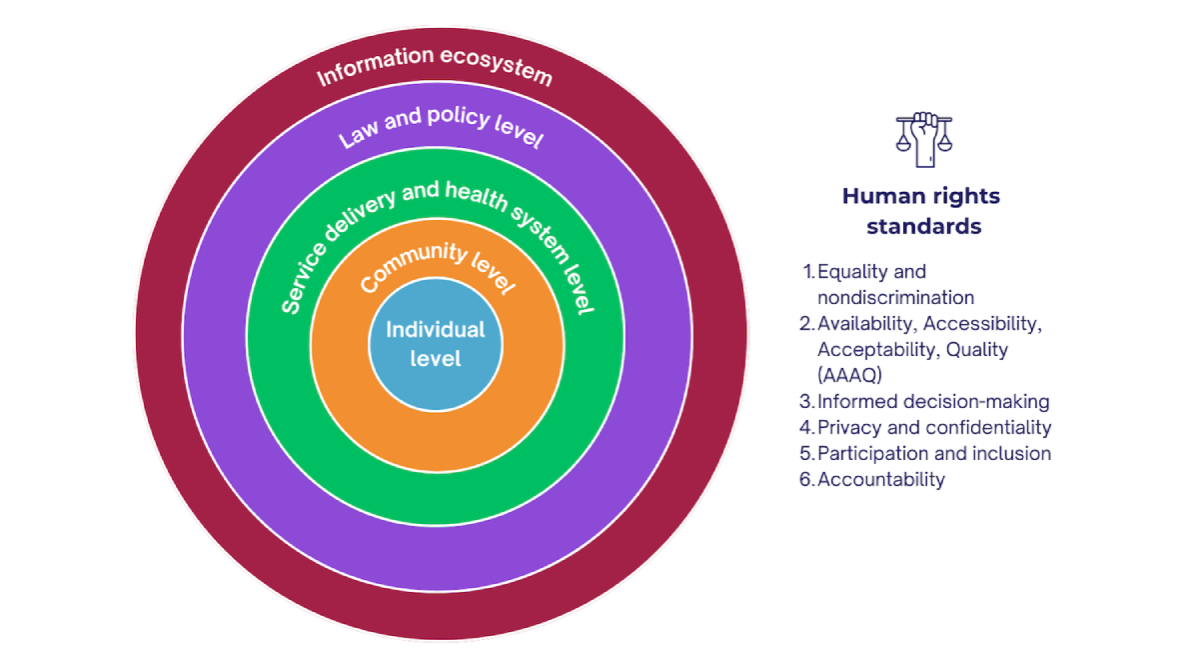
An analytical framework used in the analysis of misinformation impacts on human rights protection (reducing harms) and promotion (enabling autonomy) in the digital information environment. AAAQ: Availability, Accessibility, Acceptability, and Quality.

The results reported below used an analysis framework (Figure 4) that mapped the impacts of SRHR misinformation on human rights standards across the levels of the socioecological model. Organized by the socioecological model, 52% (n=133) studies and documents focused on the information ecosystem, followed by 15% (n=37) at the individual level, 13% (n=32) at the service delivery and health system level, 12% (n=31) at the law and policy level, and 8% (n=21) at the community level. The following sections present a mapping of the existing literature describing SRHR misinformation impacts on human rights, and reference illustrative examples from the included studies. A detailed mapping of included studies and documents by levels of the socioecological model is presented in Multimedia Appendix 5 [23,24,32-127,129-133,135-170,172-175,177-180, 182-193,195,197-213,215-218,220-236,239-249,251,252, 254-278,280,281].

#### Individual Level: Misinformation Disempowers Individual SRHR Decision-Making and Autonomy

##### Overview

The data collected through this review shows how SRHR misinformation directly undermines individuals’ autonomy by distorting health beliefs, shaping attitudes, and disrupting health-seeking behaviors. These effects are mediated by cognitive biases, social influences, and disparities in digital access and literacy. The human rights standard most consistently impacted by SRHR misinformation at the individual level is the right to informed decision-making. Impacts on access to acceptable and quality health services, and equality and nondiscrimination, particularly among youth, people of color, LGBTQ+ individuals, and people in conservative settings, were also documented. While the impact on accountabilities for human rights protection is less directly visible at the individual level, it may arise when platforms and systems fail to protect users from targeted misinformation.

##### Misleading Information Online Limits Individuals’ Ability to Make Autonomous, Informed SRHR Decisions, Exacerbated by Increasing Reliance on Digital Sources for Health Information

Misinformation online undermines individuals’ ability to make autonomous, informed SRHR decisions, especially as reliance on digital sources grows. People seeking information about contraception, fertility, abortion, or sexual health frequently encounter misleading content that influences beliefs and behaviors [32-34]. This can cause confusion and anxiety, particularly during public health crises [35]. For example, while 61% of Dutch women use pregnancy and childbirth apps, these were seen as less trustworthy than professional advice [36]. In Brazil, many new mothers use the internet to verify or replace provider guidance [37], and in Italy, 76% of women with endometriosis reported encountering online misinformation about their condition [38,39]. Similarly, 55% of men surveyed at an outpatient urology clinic in Canada reported searching online for information about men’s SRH and expressed concern about the pervasiveness of misinformation in the digital information environment [40]. Exposure to misinformation can also reinforce existing beliefs; one study found that inaccurate beliefs about fetal pain were linked with antiabortion views, shaping attitudes toward access and policy [41].

##### Misinformation Exacerbates Digital Inequities and Perpetuates Distrust, Fear, Stigma, Discrimination, and False Narratives

Studies show how digital misinformation amplifies stigma and discrimination, particularly for groups experiencing social exclusion, deterring individuals from seeking care. LGBTQ+ individuals, people of color, and those from lower-income backgrounds face higher exposure to misinformation, reinforcing existing disparities in SRHR knowledge and access [42-44]. One study described how misinformation campaigns on social media about Tenofovir-based pre-exposure prophylaxis have contributed to mistrust and hesitancy among Black, Latinx, and multiracial individuals, many of whom began to question the safety of pre-exposure prophylaxis after viewing targeted legal advertisements [44]. Similarly, social media discourse has fueled “hormonophobia” and skepticism toward hormonal contraception, often promoting unsubstantiated claims and emphasizing side effects over benefits—especially among women of color, exacerbating distrust in health care providers, contributing to vaccine hesitancy, skepticism toward contraceptives, and fear-based narratives about SRH [45].

##### Lack of Digital Access, Insufficient Health, Media, and Digital Literacy, and Lack of Protections Against Online Abuse and Fraud Make Individuals Vulnerable to Misinformation

Limited digital, information, media, and health literacy and health care access worsen misinformation exposure, leaving individuals vulnerable to scams, misinformation-driven health decisions, and fraudulent SRHR products. Women seeking fertility information online often encounter misinformation, particularly in non-Western digital spaces, leading to confusion and poor health choices [46,47]. Scammers exploit individuals seeking reproductive health solutions, as seen in Nigeria, where fraudulent “miracle fertility treatments” exploit vulnerable women with deceptive promises and financial scams [280].

#### Community Level: Misinformation Reinforces Stigma, Silencing, and Resistance to SRHR

##### Overview

The review shows that misinformation at the community level reinforces harmful social norms, perpetuates stigma, and contributes to the exclusion of marginalized voices. Human rights standards related to equality and nondiscrimination are routinely impacted, particularly when gender stereotypes, religious ideologies, or cultural beliefs are used to delegitimize SRHR. Access to quality health services and information and informed decision-making are compromised by peer-driven misinformation, while participation and inclusion are weakened when misinformation silences dissent or misrepresents marginalized populations. Furthermore, impacts on human rights accountability remain an implicit concern where misinformation is amplified by community leaders, influencers, or unregulated local media.

##### Reinforcement of Harmful Gender and Social Norms

The mapped evidence shows how misinformation perpetuates stigma against SRHR services and marginalized identities, discouraging individuals from seeking care or engaging in public discourse. Worldwide, women and girls report concerns about navigating misleading SRHR information online, in particular in spaces where misogynistic and anti-LGBTQ+ rhetoric thrives [48]. LGBTQ+ individuals face heightened misinformation exposure, particularly regarding fertility, contraception, and HIV prevention, which influences community norms and limits health care access [42-44,49]. For instance, a study in Aotearoa New Zealand found that WSW are significantly less likely to engage in cervical screening due to a legacy of misinformation about their cancer risk, reinforced by heteronormative public health messaging and provider assumptions that exclude their sexual histories [49]. 

##### Spread of Misinformation Within Communities Leads to Discriminatory Behaviors or Resistance to Evidence-Based SRHR Practices

Community-based misinformation shapes SRHR choices and can lead to harmful health behaviors. Antivaccine narratives in online parenting communities have influenced vaccine refusal, shaping norms that undermine public health efforts [50]. In South Africa and Southeast Asia, youth report misinformation, stigma, and intergenerational barriers as major obstacles to accessing SRHR services [34,51]. Online misinformation has promoted ineffective fertility-tracking methods [52,53] and also contributed to declining use of modern contraception among young people [52,53]. In Wales, for instance, uptake of the combined contraceptive pill dropped significantly between 2019 and 2023, with clinicians linking the trend to pervasive “horror stories” on TikTok and Instagram that exaggerate side effects and create fear among young women seeking care [52]*.*

##### SRHR Advocates Face Online Violence, Exclusion, and Disinformation Campaigns

SRHR misinformation fuels harassment, exclusion, and violence against individuals and organizations advocating for sexual and reproductive rights. Reproductive justice organizations face content suppression, misinformation-driven backlash, and barriers in digital advocacy [54-56]. Antirights groups actively weaponize misinformation to delegitimize SRHR movements, as seen in Kenya and Ethiopia, where foreign-funded campaigns influenced policy decisions and public discourse [57,58]. Gendered disinformation and digital violence disproportionately target women and LGBTQ+ advocates, discouraging their participation in public life [59,60]. Deepfake revenge pornography and manipulated digital content have been used to spread false narratives and intimidate SRHR advocates [60]. Gendered disinformation legitimizes violence against women and LGBTQ+ individuals while distorting discussions on gender and reproductive rights [59].

#### Service Delivery and Health System Level: Misinformation Degrades Trust, Service Delivery, and Health Worker Roles in SRHR

##### Overview

Misinformation at the service delivery and health system level undermines the quality and equity of SRHR care. It influences provider knowledge, patient expectations, and institutional practices, compromising the AAAQ of services and information. Informed decision-making is eroded when patients rely on misinformation or when providers themselves disseminate false claims. Equality and nondiscrimination are implicated when misinformation leads to substandard care for marginalized groups. Impacts on privacy and confidentiality are underdiscussed in the literature we reviewed, but are relevant, especially when digital health tools compromise user data.

##### Health Workers May Disseminate or Be Influenced by Misinformation, Contributing to Biased or Low-Quality Care

Health care providers are increasingly confronted with responding to misinformation from patients, impacting clinical decision-making and patient trust. In Australia and the United States, doctors report an increase in patients not choosing contraception when offered, requesting unnecessary procedures, or expressing concerns about reproductive health due to misinformation spread on social media [52,53,61]. Providers themselves are not immune to misinformation, as seen in deceptive marketing targeting men’s health clinics, fertility treatments, and aesthetic gynecological procedures [62-65]. For example, an investigation into direct-to-consumer men’s health clinics in the United States found widespread promotion of low testosterone treatments and erectile dysfunction therapies, often unsupported by robust evidence, and noted that clinics rarely disclosed provider credentials, contributing to potential misinformation and compromised patient safety [65]. Similarly, several studies have shown that fertility clinics routinely market unproven IVF “add-ons,” such as embryo glue or endometrial scratching, often presenting them as effective despite limited or no supporting evidence [62,64]. Additionally, some health care professionals share misinformation online, blurring professional boundaries and undermining public trust in medical expertise [66].

##### Misinformation Can Prevent or Disrupt the Delivery of SRHR Goods and Services

SRHR misinformation creates barriers to the provision of evidence-based services, particularly for contraception, abortion, and vaccines. Misinformation about (HPV) vaccines, abortion safety, and fertility treatments affects patient decision-making and reduces uptake of essential SRH services [33,67,68]. In Wales and the United States, declining contraceptive use has been linked to misinformation on TikTok and Instagram promoting unreliable fertility-tracking methods [52,53]. In a hospital in Canada, internal policies have restricted access to abortion-related websites, limiting providers’ ability to offer full referral pathways for patients [69].

##### Barriers to Equitable and Accessible Service Provision Arise From Misinformation About Health Systems or Workers

Misinformation fosters distrust in health care providers and discourages care-seeking behaviors, particularly among marginalized populations. Pregnant women, individuals with abnormal cervical screening results, and those seeking abortion services report turning to online sources due to fear of provider judgment or legal uncertainty—only to encounter further misinformation that heightens anxiety and confusion [70-72]. Antichoice narratives and misinformation about fetal tissue have shaped patient expectations and fueled clinic harassment, making it harder for providers to deliver compassionate, evidence-based care [73]. Additionally, health workers face online harassment and disinformation campaigns, further discouraging their active engagement in SRHR service provision and public education [74-77].

#### Law and Policy Level: Misinformation Is a Legal and Policy Tool for Eroding SRHR Protections

##### Overview

At the law and policy level, the review shows that misinformation can act as a mechanism for institutionalizing discrimination and rolling back SRHR protections. It shapes legal frameworks, drives policy enforcement decisions, and creates ambiguity that undermines informed decision-making and access to acceptable and quality health services. Most findings implicate governments’ human rights accountabilities, revealing weak regulation, misuse of science, and institutional complicity in the spread of disinformation. Equality and nondiscrimination and privacy, and confidentiality are routinely compromised through discriminatory laws and digital surveillance. While human rights standards related to participation and inclusion are less emphasized, misinformation suppresses engagement and discussion across differences and stifles civil society advocacy.

##### Legal Manipulation and Policy Distortion Through Misinformation

Misinformation influences the creation or perpetuation of discriminatory laws. False narratives shape restrictive SRHR policies by misrepresenting scientific evidence and swaying public opinion. In the United States, misinformation about late-term abortions and abortion pills has influenced state-level restrictions, despite FDA (Food and Drug Administration) assurances of their safety [78]. Similarly, Ghana’s anti-LGBTQ+ bill has been fueled by foreign right-wing disinformation campaigns that mischaracterize reproductive rights and LGBTQ+ advocacy as threats to society [58].

Policy decisions based on misinformation also harm equitable SRHR access. Misinformation has justified restrictive SRHR policies that limit access to essential health care. In Nigeria, false claims about how SRHR issues were integrated in the Samoa Agreement led to a national political crisis despite official debunking efforts [79]. Similarly, US laws restricting emergency contraception access have been shaped by misinformation falsely equating the emergency contraception with abortion [80].

Misinformation may also prevent effective implementation of laws and policies designed to promote and protect SRHR through health systems. Legal uncertainty and misinformation discourage providers from delivering SRHR services, even where laws permit them. Unclear abortion laws in the United States have created confusion, leading to provider reluctance to offer legally available care [78]. In Canada, a Catholic hospital restricted provider access to abortion-related websites, limiting their ability to offer comprehensive referrals [69].

In the United States, the Project 2025 playbook outlines strategies to embed misinformation into federal governance by altering agency mandates and rewording policies to stigmatize and delegitimize SRH [81]. These tactics aim to institutionalize SRHR misinformation, making it harder for the public to access accurate and credible information about abortion and related services [81].

##### Surveillance, Privacy, and Targeting of SRHR Users and Advocates

Private digital user data from tools, platforms, and devices has been used for surveillance, the spread of misinformation, and harassment related to SRH. Digital surveillance tools have been used against individuals seeking SRHR services. Antichoice activists and law enforcement agencies in the United States have used private digital user data, geofencing, and web histories to track abortion seekers and misdirect them to crisis pregnancy centers [82,83]. LGBTQ+ users have also faced privacy risks, as seen when Grindr (Grindr LLC) was labeled a national security threat due to concerns over foreign intelligence misuse of user data, without broader protections for LGBTQ+ digital privacy [84].

##### Barriers to Advocacy and Accountability

Efforts to counter SRHR misinformation are frequently obstructed by platform restrictions and targeted attacks on reproductive rights advocates. Reproductive justice organizations struggle to promote accurate information due to advertising restrictions on social media platforms, limiting outreach efforts [55]. Human rights defenders advocating for SRHR face harassment, smear campaigns, and violence, further discouraging public discourse on reproductive rights [85].

Misinformation may distort human rights accountability systems, affecting justice outcomes for those claiming their sexual and reproductive rights. Misinformation undermines evidence-based policymaking and accountability mechanisms. In the United States, flawed research linking abortion to mental health risks—despite being widely discredited—has been cited in legal cases to justify abortion restrictions [86]. Gendered misinformation also weakens legal protections against online violence, as existing laws often fail to address digital threats and harassment effectively [59].

#### Information Ecosystem Level: Information Ecosystem Design Systemically Undermines SRHR Rights and Equity

##### Overview

This review reveals that the information ecosystem, comprising social media, search engines, digital ads, algorithms, mobile apps, AI agents, other online spaces, and platform governance, is a key enabler of systemic SRHR misinformation. Platform design choices and business models allow misinformation to flourish while silencing accurate, rights-based content. This results in impacts across all six categories of human rights standards: algorithmic bias reinforces discrimination, censorship of accurate SRHR content limits AAAQ, exposure to misleading content undermines informed decision-making, tracking and surveillance violate privacy, content moderation suppresses participation, and regulatory inaction weakens accountability.

##### Platform Architecture and Digital Business Models Enable Harm From SRHR Misinformation

Misinformation distorts public discourse, crowds out accurate information, and undermines trust in credible, accurate SRHR information and sources. SRHR misinformation spreads rapidly in high-engagement digital spaces, reinforcing stigma, confusion, and distrust in public health sources. During the 2022 mpox outbreak, social media misinformation fueled stigma against sexual minority men and gender-diverse individuals, reinforcing discrimination [87].

SRH misinformation has been monetized in an unregulated digital information ecosystem, undermining consumers’ ability to make informed choices. Platforms, influencers, and wellness brands profit from misleading SRHR narratives, making it harder for individuals to access evidence-based guidance. YouTube, TikTok, and Instagram influencers promote unregulated fertility apps, alternative contraceptive methods, and nonevidence-based treatments for polycystic ovary syndrome and erectile dysfunction [88-92]. Google and Meta profit from deceptive advertising, directing users searching for abortion services to crisis pregnancy centers that provide misleading information [93].

Vague and inconsistently enforced content moderation policies enable misinformation while restricting SRHR advocacy. Social media platforms fail to regulate misinformation effectively while disproportionately censoring SRHR content. Meta restricts advertising by abortion providers while allowing antichoice misinformation to spread unchecked [93]. Google search results for abortion medication often lead to misleading crisis pregnancy centers [94]. Additionally, retail platforms such as Amazon (Amazon.com, Inc) have been reported to restrict or delist vaginal health products—such as therapeutic devices for pelvic pain—labeling them “potentially embarrassing,” thereby limiting access to essential women’s health products and reinforcing stigma around SRH care [282].

##### Inequities in SRHR Content Access and Algorithmic Asymmetries and Discrimination

Inequalities in digital access, the design of internet platforms, and digital marketing tools lead to uneven exposure to accurate SRHR information. Algorithmic bias and content moderation disparities create unequal access to accurate SRHR information. Platforms inconsistently apply moderation policies, disproportionately censoring sexual health content while allowing misinformation to flourish, particularly in low-income countries [95-97]. In Egypt and Western Asia, SRHR advocates face content suppression, including blocked ads and limited reach [56].

The quality of SRHR health information online is low, with misinformation incidence rates ranging from 14% to 97% across topics and platforms [98-119]. Studies show 41% of Facebook articles about HPV vaccination contained misinformation [115], 28% of YouTube videos about erectile dysfunction included misleading content [102], and 73% of TikTok videos about gynecological cancers were of poor quality [99].

##### Global Flow and Amplification Mechanisms of Misinformation

Digital misinformation on SRHR easily transcends national boundaries, affecting global health policies and legislation. Far-right groups in Europe have replicated US misinformation campaigns, targeting LGBTQ+ rights and reproductive health access [120]. In Kenya, a far-right group manipulated X (formerly known as Twitter) discussions to derail support for reproductive health bills [121,122].

Triggers of misinformation surges include public events, sensationalized news, and humor. Misinformation spikes during public health emergencies and policy changes. The reversal of Roe versus Wade triggered an abortion-related infodemic, where evolving laws, social media misinformation, and inadequate content moderation fueled confusion [94,123]. News coverage of contraceptive lawsuits has exaggerated risks, shaping global perceptions and fueling misinformation [124]. Additionally, a rising antirights movement in Ethiopia, aligned with the US Christian Right, is working to dismantle the right to safe and legal abortion, propagating SRH misinformation online, all the while gaining influence in political spaces [57].

Drivers of misinformation amplification in the digital information environment include scams, memes, clickbait, and marketing. SRHR misinformation spreads through viral content, influencer marketing, and deceptive digital campaigns. Scammers posing as fertility experts in Nigeria have exploited vulnerable women with false “miracle treatments” [280]. Another emerging form of online harm involves “Munchausen by internet,” where individuals fabricate perinatal illnesses and crises in real time—such as neonatal death—using fake online personas [125]. These cases can emotionally manipulate and mislead health care providers, wasting their time and creating distress when the deception is uncovered [125]. In addition, memes on TikTok are used to evade content moderation while spreading misinformation about contraception and sexual health [126].

Lack of regulation or accountability for digital health misinformation undermines public health efforts. SRHR misinformation remains largely unregulated, enabling bad actors to shape digital discourse. WHO has identified AI-generated misinformation as a growing threat to SRHR, as AI tools produce misleading reproductive health content with little oversight [23]. Efforts to counter misinformation are often obstructed by digital platform policies, limiting reproductive justice advocacy [55].

## Discussion

### Principal Findings

This scoping review maps and describes existing literature on misinformation in SRHRs, suggesting that it is rarely limited to isolated myths, individual misunderstandings, or politicized topics. Instead, the evidence reviewed points to SRHR misinformation as being entangled with the broader social, political, and digital systems through which health information is searched for, interpreted, shared, and acted upon. Traditional responses to health misinformation have often focused on correcting false beliefs through education, counter-messaging, or fact-checking, which are based on communication frameworks and exclude systems thinking and information environment, approaches, and analysis through human rights standards. The literature reviewed highlights concerns that such approaches may be insufficient, particularly given the disproportionate impacts on underserved communities. Several studies suggest that SRHR misinformation can operate within wider structures of inequality, stigma, and power, and in some cases, may be deliberately used to undermine reproductive autonomy, challenge evidence-based care, or reinforce discriminatory ideologies.

This scoping review focused on the five years since 2019 specifically because the digital information environment, architectures, algorithms, platform moderation policies, as well as socioeconomic dynamics have been changing rapidly, also in relation to how we understand misinformation and the wider impacts of the information environment on health behaviors. Capturing the recent experience of users seeking health information, support, and care in digital spaces more closely reflects the digital realities of today. During this time, users online have experienced a shift to reduced moderation on many large platforms [26,283] and related polarization of conversations in digital spaces [284,285], the impacts of the pandemic on straining health systems health promotion programs and their capacity to address the information environment [20], as well as rapidly evolving ways users seek information, support and companionship through synthetic companions and AI-powered tools and interfaces. The themes coming out of this review show that this changing sociotechnical landscape is both concentrating harms in groups that seek SRHR-related support and care online, and at the same time, the features of the information environment are used by communities to exercise autonomy in relation to how they access, discuss, and act on health information.

These insights are aligned with the growing shift in the broader health misinformation research and practice field, where scholars have called for moving beyond individual-level interventions to engage with the systemic, emotional, and relational dynamics that shape how people encounter and respond to information. The results of the mapping underscore that SRHR misinformation is not an exception but a critical and often overlooked example of these wider dynamics.

This paper is the first of three planned publications based on this scoping review’s dataset and focuses on cross-mapping information science and misinformation research with human rights principles. The overall purpose of this work is to bring the research and advocacy community across different fields together to improve human rights protection and promotion in the context of SRHR misinformation in digital spaces. Future papers will detail the attributes of SRHR misinformation and harm, and interventions reported in the included studies, while the third will be the result of a consultation with human rights scholars and advocates on improving concepts and monitoring frameworks of the harmful impact misinformation has on SRHR protection and promotion.

### What Makes SRHR Misinformation Distinct

What makes SRHR misinformation distinct from misinformation in other health areas (such as vaccine-preventable diseases, cancer, or emergency preparedness and response) is its intrinsically gendered and politicized nature. SRHR is deeply connected to individual autonomy, social identity, and cultural norms. Misinformation in this space is not only shaped by stigma and taboo, but it often also reinforces them. It is commonly weaponized to control reproductive choices, restrict access to care, and limit public discourse on gender and sexuality. Unlike many other health domains, SRHR misinformation often targets and impacts people differently based on their gender identity, sexual orientation, reproductive status, or age, reinforcing longstanding power imbalances.

Increasingly in most contexts, the search for SRHR information begins and ends online. Many individuals, particularly those in marginalized or stigmatized communities, seek answers to sensitive questions via the likes of search engines, mobile apps, social media, AI agents and tools, and private messaging platforms. Due to its intimate, gendered, and relational nature, SRHR misinformation does not just distort facts. It also shapes how people understand their rights, identities, and relationships. These characteristics make SRHR topics particularly vulnerable to disinformation, where individuals, influencers, or organizations deliberately spread misleading narratives or promote unproven products and services for financial gain or political influence.

Addressing the harm from SRHR misinformation, therefore, goes beyond correcting content or narratives and requires confronting and transforming the systemic drivers, commercial incentives, and sociopolitical norms that produce and legitimize harmful information about SRHR.

### Terminological and Conceptual Inconsistencies

The analysis in Figure 2 reflects what kinds of contextualization and value judgments are placed on the concept of misinformation in the studies included in this review. Importantly, this review reveals a wide diversity in the terminology used to describe SRHR misinformation. While the most common terms used are “misinformation” and “inaccurate,” the rest of the terms used all point to an attribute or impact of information itself, not merely its accuracy. Terms such as “misleading,” “inappropriate,” “unreliable,” “gendered disinformation,” “myths,” “incomplete,” and “harmful narratives” were used across the studies. This suggests that authors from different disciplines (eg, public health, law, or media studies) and professional groups (eg, clinicians, health educators, policy analysts, or advocates) differently interpret and frame the qualities of information, such as its accuracy, relevance, intent, and social impact. This might stem from the drivers and harms perceived as most relevant to SRHR. These conceptual differences likely shape how misinformation is approached in the literature and by advocates and may present challenges for synthesizing evidence, developing effective interventions, and creating shared standards across health topics and research disciplines. Future research must grapple with this definitional pluralism and embrace cross-disciplinary approaches that can accommodate and bridge these varied perspectives. One approach to acknowledge this diversity is to move from discussing “misinformation” to studying “information environment,” as many of the drivers and impacts, including harms, described in this paper are related to social, cognitive, and other drivers of how information is interpreted and meaning is made, rather than the accuracy of a piece of information alone.

These terminological inconsistencies also reflect a deeper fragmentation in the research, beyond language, regarding how the problem of SRHR misinformation is conceptualized and situated within broader systems. This scoping review shows that while there is growing recognition that SRHR misinformation is a public health issue, the literature remains fragmented. Most of the studies identified in this review focus on describing or measuring misinformation in specific domains or platforms (such as abortion or contraception, YouTube or X [formerly known as Twitter]), with fewer addressing the broader political, cultural, and systemic conditions that shape the production, circulation, and impact of misinformation in SRHR. This pattern mirrors wider critiques in the health misinformation research field, which caution against overly narrow, content-focused approaches that overlook the complexity of digital ecologies and information environments. As recent scholarship has argued, health misinformation is not simply a matter of individual belief or factual inaccuracy, but a relational and evolving process shaped by identity, emotion, trust, and social context [2,3,14]. Our findings underscore the need for SRHR misinformation research to engage more fully with these broader frameworks to inform more effective, equity-oriented responses.

### Human Rights Framework and Structural Harms

A key contribution of this review is the application of a human rights framework to map the harms of SRHR misinformation across the socioecological model. We found that misinformation impacts multiple human rights standards, including informed decision-making, equality and nondiscrimination, availability and quality of information, services and goods, privacy, participation, and accountability. These violations occur at different levels, undermining individual autonomy, fueling stigma within communities, disrupting evidence-based service delivery, enabling discriminatory laws and population surveillance, and fostering unregulated information ecosystems that prioritize virality over veracity. This lens clarifies how SRHR misinformation is not merely a matter of faulty information, but rather that it is a matter of human rights, social justice, and systemic inequity.

Notably, this review highlights a gap in current SRHR and human rights literature and practice—while the right to SRH has long emphasized access to accurate information, it has yet to fully grapple with how information ecosystems—including ecosystem architecture, algorithmic governance, and commercial content moderation and marketing tools [24,26]—function as structural determinants of health. Digital environments now shape not only what information is available, but whose voices are amplified, whose experiences are silenced, and whose health is protected or compromised [2,14]. The failure to regulate or even acknowledge these dynamics constitutes a failure of accountability [24,26].

### Implications for Research, Policy, and Practice

Understanding misinformation as ecosystemic has major implications for public health, policy, and advocacy. Health systems can no longer treat misinformation solely as a public education issue and must engage with how SRHR information is designed, curated, and circulated across digital infrastructures. This requires a shift from short-term, message-based communication and reactive debunking to proactive governance that supports digital literacy, designs inclusive information pathways, and builds social cohesion and institutional trust. SRHR advocates that communities, especially marginalized communities, must be protected from online harassment and supported to participate meaningfully in digital public spheres.

Policy makers must reaffirm access to accurate, evidence-based health information as a public good and a fundamental human right, rather than a consumer product vulnerable to manipulation. Regulatory frameworks should ensure transparency in platform algorithms, protection of privacy, and equitable access to information. Meanwhile, digital platforms must be held accountable for the role they play in facilitating and profiting from SRHR misinformation. Monitoring and addressing these harms will require collaborative efforts across sectors, including health, education, technology, and law, and must center the voices of those most affected by SRHR misinformation and information injustice.

This therefore points to several research priorities in this space. First, there is a need to move beyond cataloguing misinformation content to studying how SRHR misinformation functions within broader digital ecosystems—not only how it spreads, why it resonates with particular communities, and how trust in different sources is constructed, but also what are the “dark patterns” that propagate vulnerabilities of SRHR, the commercial determinants of SRHR misinformation, and the evolving relationships with information online that discard its tactfulness for relatability. Second, future work should explore how individuals and groups resist, reinterpret, or correct misinformation, particularly in marginalized communities, and what forms of digital literacy and social support strengthen resilience. Third, more research is needed into the interactions between misinformation, digital platform design, and regulatory policies, including how algorithmic amplification works, how the use of private user data has been monetized, and content moderation affect health-related rights. Finally, interdisciplinary inquiry should investigate how to embed human rights frameworks into technology governance, including the development of accountability indicators that assess whether information environments support or hinder the right to SRHR. These research directions are essential to inform rights-based, equity-driven strategies to counter SRHR misinformation at scale.

### Limitations

This scoping review has several limitations. First, our inclusion criteria restricted the search to English-language sources, which may have excluded relevant studies published in other languages. Second, by focusing exclusively on the digital information environment, we did not capture insights from offline spaces or additional media channels where SRHR misinformation might also circulate and impact communities. Third, we included only studies and documents explicitly addressing misinformation, and this approach may have overlooked research that does not focus on misinformation but on other challenges within the information ecosystem that share drivers, amplifiers, and attributes with misinformation, such as questions, concerns, information voids, hate speech, or disinformation (for promoting ideas or products for profit or influence).

### Conclusions

The consequences of SRHR misinformation in the digital information environment include restricted bodily autonomy, compromised reproductive justice, and distorted policy environments. SRHR misinformation includes a wide spectrum of topics, platforms, and various effects and harms that infringe on human rights principles and standards. This scoping review described the multitudinous social, technological, and regulatory drivers that shape discourse about health online and the many ways individuals’ SRH and human rights are affected by it online and offline. No single level of the socioecological model is sufficient to describe the scope and effects of SRHR misinformation in the digital information environment, and solutions are also unlikely to be found at a single level.

Although this scoping review cataloged some of the effects and harms of SRHR misinformation, fuller accounting is needed to understand the cumulative effects and harms of SRHR misinformation in individuals, communities, health systems, and societies. Inspiration can be drawn from more established fields of health misinformation and behavioral science research in vaccines, cancer, and pandemic preparedness, where misinformation is a common risk to health care decisions and behaviors, and has been long studied, and impacts have been well-catalogued.

The internet will only become more ingrained in the day-to-day lives of people and their families and communities, and so, the SRHR field must evolve with this now indispensable source of information, connection, and care to help everyone realize their right to SRHR health care everywhere, including online.

## Appendix

Multimedia Appendix 1 PRISMA-ScR checklist.

Multimedia Appendix 2 Search strategy and results of search across different databases.

Multimedia Appendix 3 Included studies and documents.

Multimedia Appendix 4 Characteristics of included studies and documents.

Multimedia Appendix 5 Studies and documents mapped based on impacts of SRHR misinformation on human rights principles by levels of the socioecological model. SRHR: sexual and reproductive health and rights.

Multimedia Appendix 6 Transcripts of ChatGPT conversations that were used to solicit editing feedback in the preparation of this manuscript.

## Abbreviations

AAAQ: Availability, Accessibility, Acceptability, and Quality
AI: artificial intelligence
FDA: Food and Drug Administration
HPV: human papillomavirus
JBI: Joanna Briggs Institute
LGBTQ+: Lesbian, Gay, Bisexual, Transgender, Queer, and more
PRISMA-ScR: Preferred Reporting Items for Systematic Reviews and Meta-Analyses extension for Scoping Reviews
SRH: sexual and reproductive health
SRHR: sexual and reproductive health and rights
UN: United Nations
WHO: World Health Organization

## Data Availability

Data is available in supplementary materials and upon reasonable request to the corresponding author.
