# Supplementary material for: Impacts of Sexual and Reproductive Health and Rights Misinformation in Digital Spaces on Human Rights Protection and Promotion: Scoping Review
Source: JMIR Infodemiology. 2025 Dec 30;5:e83747. doi: 10.2196/83747 (PMC12811040; doi:10.2196/83747)

# PUBMED

( (((sexual OR reproductive) AND (health OR rights)) OR pregnan* OR (“men’s health” OR infertility ) )

AND (misinformation OR “information provision” OR “online information”)

AND ("digital spaces" OR "social media" OR "online” OR “platform*" OR "internet" OR "TikTok" OR "Facebook" OR "YouTube" OR "Instagram" OR "Reddit" OR "blogs" OR "forums" OR "online communit*" OR “text mess*”) )

And last 5 years


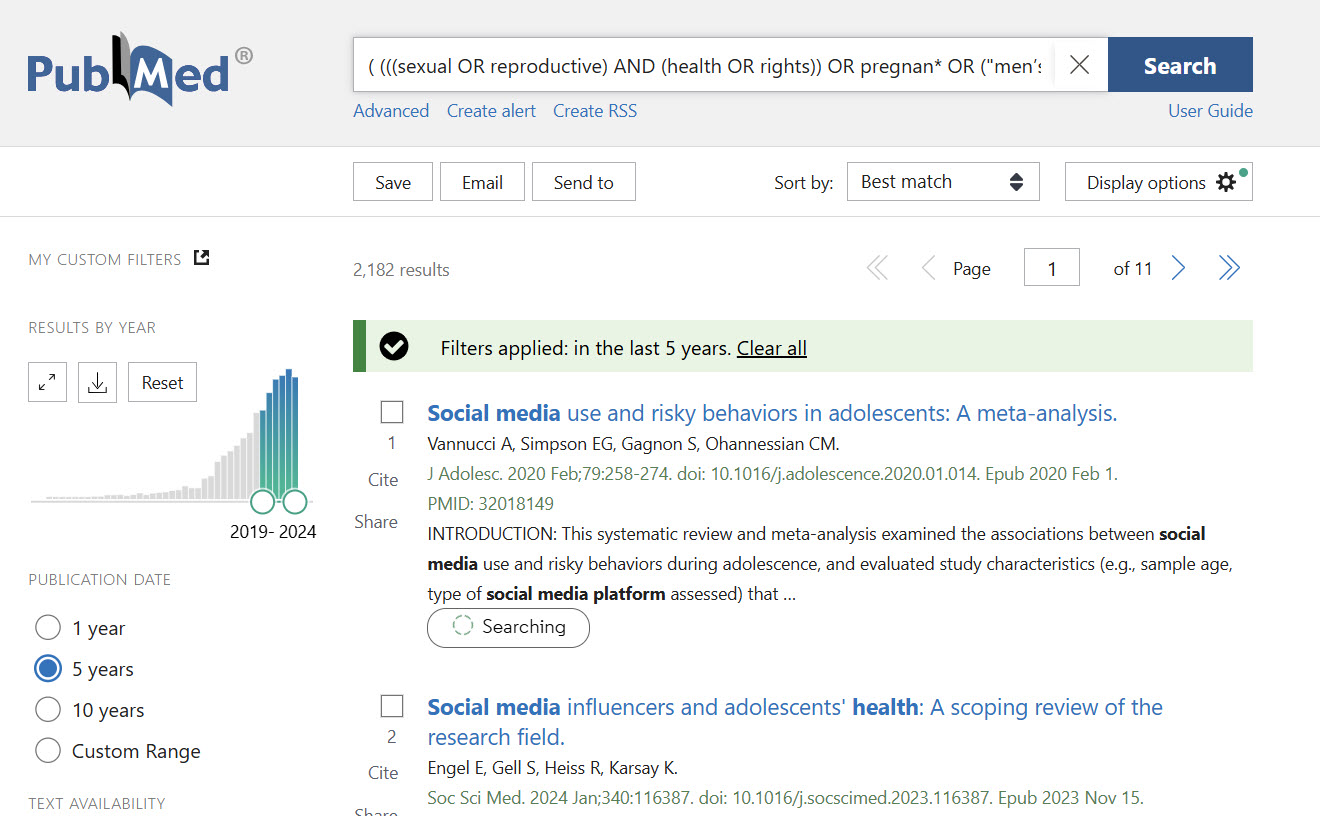


# Web of Science

TS=(

(

(

(sexual OR reproductive) AND (health OR rights)

)

OR pregnan*

OR ("men’s health" OR infertility)

)

AND

(misinformation OR "information provision" OR "online information")

AND

("digital spaces" OR "social media" OR "online" OR "platform*" OR "internet" OR "TikTok" OR "Facebook" OR "YouTube" OR "Instagram" OR "Reddit" OR "blogs" OR "forums" OR "online communit*" OR "text mess*")

)

AND PY=(2019-2024)


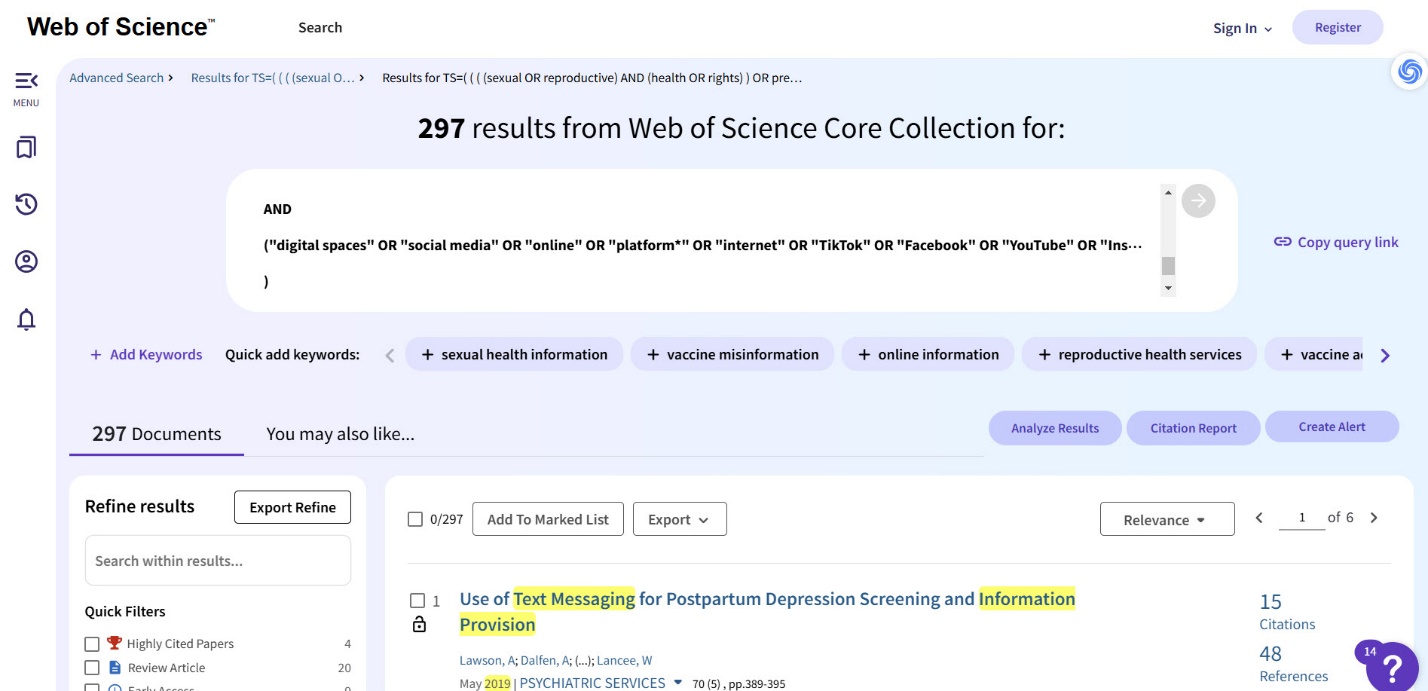


# Scopus

TITLE-ABS-KEY(

(

(sexual OR reproductive) AND (health OR rights)

OR pregnan*

OR ("men’s health" OR infertility)

)

AND

(misinformation OR "information provision" OR "online information")

AND

("digital spaces" OR "social media" OR "online" OR "platform*" OR "internet" OR "TikTok" OR "Facebook" OR "YouTube" OR "Instagram" OR "Reddit" OR "blogs" OR "forums" OR "online communit*" OR "text mess*")

)

AND PUBYEAR > 2018


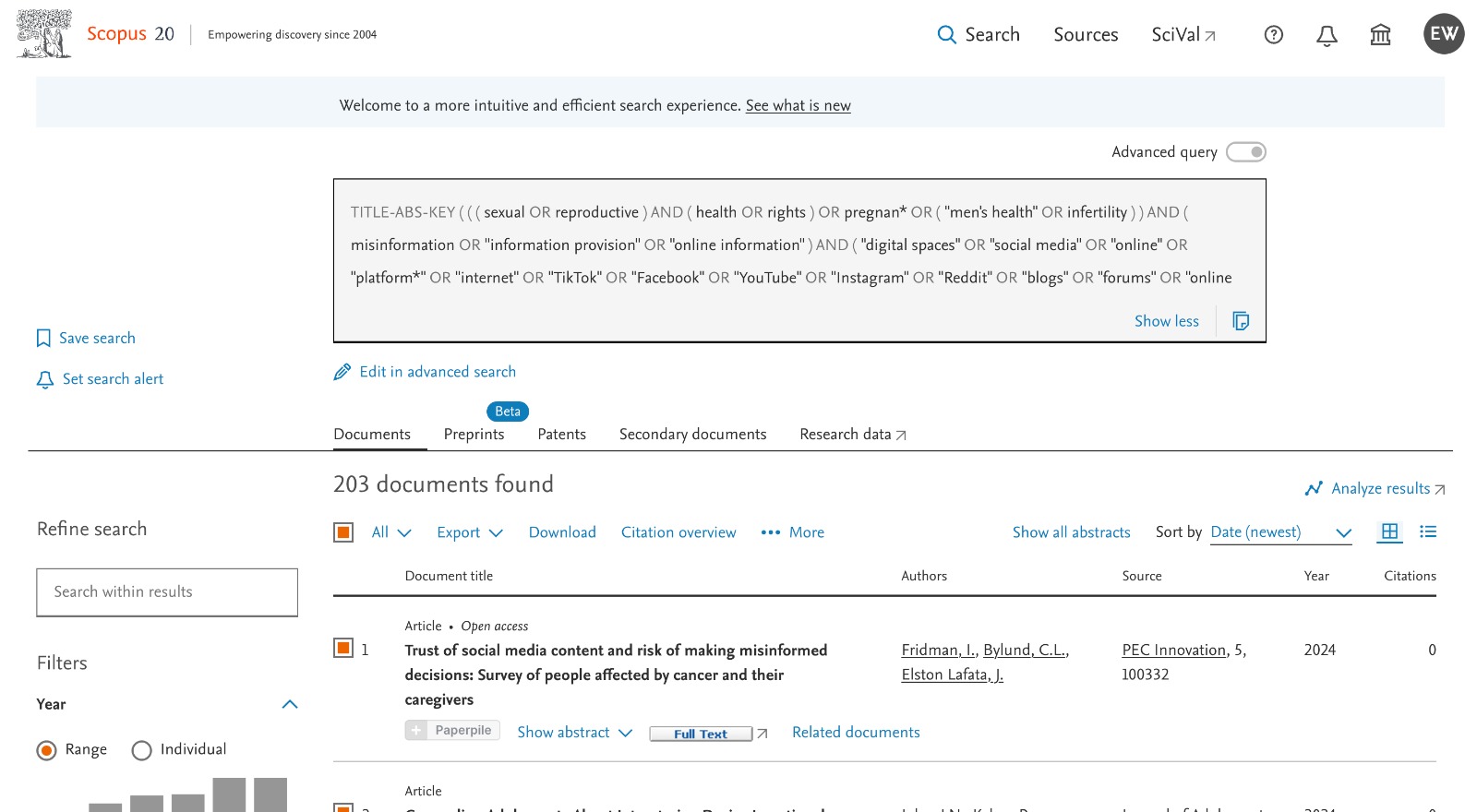


# Social Science Citation Index

- Social Science Citation Index )covered by web of science)

# JSTOR

((sexual OR reproductive) AND (health OR rights)) AND misinformation AND (online OR internet OR “social media”)

2019-2024

Journals only


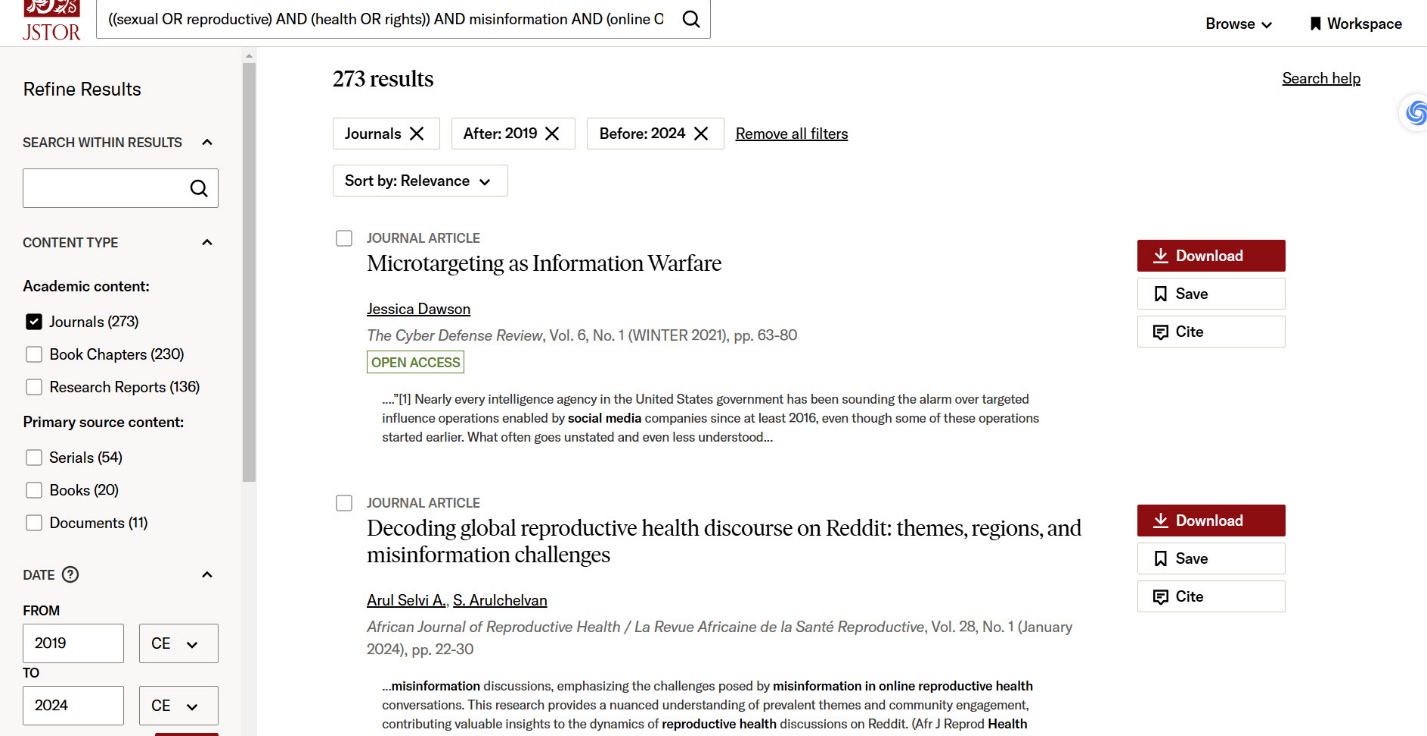


# HEIN ONLINE

catalog:(("social media" OR online) AND *information AND (reproductive OR health))

# Google scholar

((sexual OR reproductive) )

AND misinformation

AND “social media”

-review

Only 2024


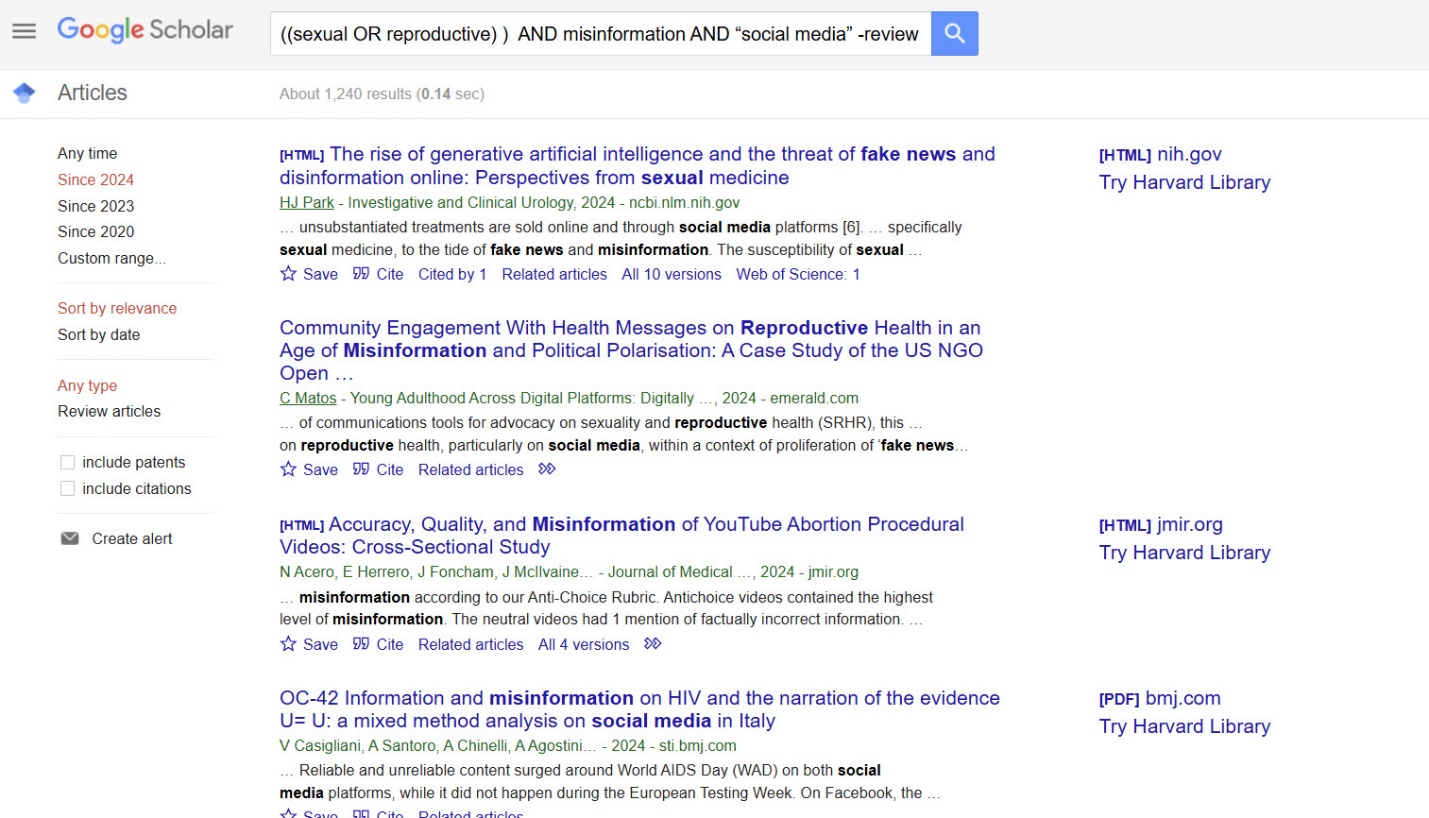

Supplement: Multimedia Appendix 2 [file infodemiology_v5i1e83747_app2.docx]
