# Supplementary material for: Impacts of Sexual and Reproductive Health and Rights Misinformation in Digital Spaces on Human Rights Protection and Promotion: Scoping Review
Source: JMIR Infodemiology. 2025 Dec 30;5:e83747. doi: 10.2196/83747 (PMC12811040; doi:10.2196/83747)
Supplement: Multimedia Appendix 3 [file infodemiology_v5i1e83747_app3.docx]

1. Acero N, Herrero E, Foncham J, McIlvaine J, Kayaalp E, Figueora M, et al. Accuracy, Quality, and Misinformation of YouTube Abortion Procedural Videos: Cross-Sectional Study. J Med Internet Res. 2024;26:e50099.
2. ACOG. Ethical Issues With Vaccination in Obstetrics and Gynecology: American College of Obstetricians and Gynecologists (ACOG) Committee Opinion, Number 829. Obstet Gynecol. 2021;138(1):e16-e23.
3. Adegoke Y, Nwonwu C, Shaikhouni L. 'Pregnant' for 15 months: Inside the 'miracle' fertility scam: BBC; 2024 [Available from: https://www.bbc.com/news/articles/c78dyryreyxo.
4. Agrawal S, Irwin C, Dhillon-Smith RK. An evaluation of the quality of online information on emergency contraception. Eur J Contracept Reprod Health Care. 2021;26(4):343-8.
5. Alam T, Baroni A, Davis SL, Faiza A, González-Uribe C, Guerrero Castro J, et al. The digital transformation and the right to health of young adults in Bangladesh and Colombia: a community-engaged study. Health and Human Rights. 2024;26(2).
6. AlHefdhi HA, Mahmood SE, Alsaeedi MAI, Alwabel HHA, Alshahrani MS, Alshehri EY, et al. COVID-19 Vaccine Uptake and Hesitancy among Pregnant and Lactating Women in Saudi Arabia. Vaccines (Basel). 2023;11(2).
7. Allison BA, Vear K, Hoopes AJ, Maslowsky J. The perceived impact of a post-Dobbs landscape on U.S. adolescents and young adults. Contraception. 2024;138:110513.
8. Anderer S. Patients are turning to TikTok for health information-here's what clinicians need to know. JAMA. 2024;331(15):1262-4.
9. Arena A, Degli Esposti E, Orsini B, Verrelli L, Rodondi G, Lenzi J, et al. The social media effect: the impact of fake news on women affected by endometriosis. A prospective observational study. Eur J Obstet Gynecol Reprod Biol. 2022;274:101-5.
10. Arul Selvi A, Arulchelvan S. Decoding global reproductive health discourse on Reddit: themes, regions, and misinformation challenges. African J Reprod Health. 2024;28(1):22-30.
11. Association CM. COMMENTARY: Combatting misinformation in women’s health 2024 [Available from: https://www.cma.ca/about-us/what-we-do/press-room/commentary-combatting-misinformation-womens-health.
12. Bal DS, Panchendrabose K, Van Iderstine MG, Patel P. The impact of misinformation on patient perceptions at a men's health clinic: a cross-sectional study. Int J Impot Res. 2024;36(6):636-40.
13. Basinger ED, Delaney AL, Williams C. Uncertainty Management in Online Sexual Health Forums. Health Commun. 2023;38(5):875-84.
14. Beilby K, Hammarberg K. ChatGPT: a reliable fertility decision-making tool? Hum Reprod. 2024;39(3):443-7.
15. Berendes S, Mounier-Jack S, Ojo-Aromokudu O, Ivory A, Tucker JD, Larson HJ, et al. "Figuring stuff out myself" - a qualitative study on maternal vaccination in socially and ethnically diverse areas in England. BMC Public Health. 2023;23(1):1408.
16. Berg S. What doctors wish patients knew about birth control: American Medical Association; 2024 [Available from: https://www.ama-assn.org/delivering-care/public-health/what-doctors-wish-patients-knew-about-birth-control.
17. Berger MN, Taba M, Marino JL, Lim MSC, Cooper SC, Lewis L, et al. Social media's role in support networks among LGBTQ adolescents: a qualitative study. Sex Health. 2021;18(5):421-31.
18. Berkowitz HE, Vann JCJ. Strategies to Address COVID-19 Vaccine and Pregnancy Myths. MCN Am J Matern Child Nurs. 2023;48(4):215-23.
19. Bernstein A, Friedrich-Karnik A, Damavandi S. How Project 2025 Seeks to Obliterate Sexual and Reproductive Health and Rights: MS. Magazine; 2025 [Available from: https://msmagazine.com/2024/10/16/project-2025-seeks-to-obliterate-sexual-and-reproductive-health-and-rights/?utm_source=chatgpt.com.
20. Bernstein A, Friedrich-Karnik A, Damavandi S. How Project 2025 Seeks to Obliterate Sexual and Reproductive Health and Rights: Guttmacher Institute; 2024 [Available from: https://www.guttmacher.org/fact-sheet/how-project-2025-seeks-obliterate-srhr.
21. Biino M. Anti-transgender campaigns bussed across the Atlantic transform European politics. Coda Story. 2024.
22. Blackburn NA, Dong W, Threats M, Barry M, LeGrand S, Hightow-Weidman LB, et al. Building Community in the HIV Online Intervention Space: Lessons From the HealthMPowerment Intervention. Health Educ Behav. 2021;48(5):604-14.
23. Boatman D, Starkey A, Acciavatti L, Jarrett Z, Allen A, Kennedy-Rea S. Using Social Listening for Digital Public Health Surveillance of Human Papillomavirus Vaccine Misinformation Online: Exploratory Study. JMIR Infodemiology. 2024;4:e54000.
24. Borrás Pérez P. Facebook Doesn't Like Sexual Health or Sexual Pleasure: Big Tech's Ambiguous Content Moderation Policies and Their Impact on the Sexual and Reproductive Health of the Youth. Int J Sex Health. 2021;33(4):550-4.
25. Bott S. Fears misinformation behind drop in contraception. BBC. 2024.
26. Bradshaw AS, Shelton SS, Wollney E, Treise D, Auguste K. Pro-Vaxxers Get Out: Anti-Vaccination Advocates Influence Undecided First-Time, Pregnant, and New Mothers on Facebook. Health Commun. 2021;36(6):693-702.
27. Brissette V, Alnaki A, Garfinkle R, Lloyd M, Demian M, Vasilevsky CA, et al. The quality, suitability, content and readability of online health-related information regarding sexual dysfunction after rectal cancer surgery. Colorectal Dis. 2021;23(2):376-83.
28. Burns C, Bakaj A, Berishaj A, Hristidis V, Deak P, Equils O. Use of Generative AI for Improving Health Literacy in Reproductive Health: Case Study. JMIR Form Res. 2024;8:e59434.
29. Caddy C, Cheong M, Lim MSC, Power R, Vogel JP, Bradfield Z, et al. "Tell us what's going on": Exploring the information needs of pregnant and post-partum women in Australia during the pandemic with 'Tweets', 'Threads', and women's views. PLoS One. 2023;18(1):e0279990.
30. Capece M, Di Giovanni A, Cirigliano L, Napolitano L, La Rocca R, Creta M, et al. YouTube as a source of information on penile prosthesis. Andrologia. 2022;54(1):e14246.
31. Çardakcı Bahar Ş, Özarslantürk S, Özcan E. Does YouTube™ Provide Adequate Information on Oral Health During Pregnancy? Cureus. 2024;16(4):e57887.
32. Cartmell KB, Mzik CR, Sundstrom BL, Luque JS, White A, Young-Pierce J. HPV Vaccination Communication Messages, Messengers, and Messaging Strategies. J Cancer Educ. 2019;34(5):1014-23.
33. Carvalho de Sousa R, Lima da Silva MJ, Fialho do Nascimento MR, da Cruz Silveira M, Fernandes FP, Quaresma TC, et al. Influences on COVID-19 Vaccine Adherence among Pregnant Women: The Role of Internet Access and Pre-Vaccination Emotions. Int J Environ Res Public Health. 2024;21(6).
34. Chaiken SR, Han L, Darney BG, Han L. Factors Associated With Perceived Trust of False Abortion Websites: Cross-sectional Online Survey. J Med Internet Res. 2021;23(4):e25323.
35. Chee RM, Capper TS, Muurlink OT. Social media influencers' impact during pregnancy and parenting: A qualitative descriptive study. Res Nurs Health. 2024;47(1):7-16.
36. Chen C, Wang X. Reporting Online Aggression: A Transnational Comparative Interface Analysis of Sina Weibo and Twitter. Tech Commun. 2023;70(4):42-59.
37. Cheng JW, Fernandez N, Shnorhavorian M, Merguerian PA, Kieran K. Engagement of common pediatric urologic conditions on social media. J Pediatr Urol. 2022;18(2):236.e1-.e7.
38. Chervenak J, Lieman H, Blanco-Breindel M, Jindal S. The promise and peril of using a large language model to obtain clinical information: ChatGPT performs strongly as a fertility counseling tool with limitations. Fertil Steril. 2023;120(3 Pt 2):575-83.
39. Chinogwenya W. The fight for reproductive rights has gone digital – platforms like Meta and Google must pick a side. The Telegraph. 2024.
40. Choices CfCDHMR. Digital Disparities: The global battle for reproductive rights on social media. 2024.
41. Cilio S, Collà Ruvolo C, Turco C, Creta M, Capece M, La Rocca R, et al. Analysis of quality information provided by "Dr. YouTube(TM)" on Phimosis. Int J Impot Res. 2023;35(4):398-403.
42. Cohen C, Serrano L. Anti-Abortion Centers in California in 2023: Number, State Licensure, Location, and False Medical Claims Online. UCLA Center on Reproductive Health, Law, and Policy; 2024.
43. Collà Ruvolo C, Califano G, Tuccillo A, Tolentino S, Cancelliere E, Di Bello F, et al. "YouTube™ as a source of information on placenta accreta: A quality analysis". Eur J Obstet Gynecol Reprod Biol. 2022;272:82-7.
44. Cooke-Jackson A, Rubinsky V, Gunning JN. "Wish I Would Have Known that before I Started Using It": Contraceptive Messages and Information Seeking among Young Women. Health Commun. 2023;38(4):834-43.
45. Côté EJM, Benton M, Gardner R, Tribe R. Balancing benefits and risks of exercise in pregnancy: a qualitative analysis of social media discussion. BMJ Open Sport Exerc Med. 2024;10(4):e002176.
46. D’Angelo SM, Marcus R, Khan A, Homonchuk O. Whose hands on our education? Identifying and countering gender-restrictive backlash. Align Platform; 2024.
47. Dalton ME, Duffy R, Quinn E, Larsen K, Peters C, Brenner D, et al. A qualitative review of social media sharing and the 2022 monkeypox outbreak: did early labelling help to curb misinformation or fuel the fire? Sex Health. 2024;21.
48. Davey M. Companies marketing useless health products to women using feminist wellbeing messages: The Guardian; 2024 [Available from: https://www.theguardian.com/australia-news/2024/feb/15/companies-marketing-useless-health-products-to-women-using-feminist-wellbeing-messages.
49. Davies M. Row over medical journal’s refusal to retract paper used to restrict abortion in US legal cases. The BMJ. 2023(382):1576.
50. Dawson AC, Fitzpatrick AK, Matthews JM, Nguyen AAK, Papanaoum K, Smith JR. Characterisation of social media conversations on syphilis: an unobtrusive observational study. Sex Health. 2024;21.
51. Demirci A, Başar H. Youtube is an unreliable source of information about delayed ejaculation treatment. Urologia. 2024;91(2):403-12.
52. Di Spirito F, Amato A, D'Ambrosio F, Cannatà D, Di Palo MP, Coppola N, et al. HPV-Related Oral Lesions: YouTube Videos Suitability for Preventive Interventions including Mass-Reach Health Communication and Promotion of HPV Vaccination. Int J Environ Res Public Health. 2023;20(11).
53. Dietrich PN, Doolittle J, Brink S, Hanna D, Fitzgerald J, Dadhich P, et al. An Online Investigation Into Direct-to-Consumer Men's Health Clinics: The Who, What, and Where. Urology. 2023;174:135-40.
54. Diez SL, Fava NM, Fernandez SB, Mendel WE. Sexual health education: the untapped and unmeasured potential of US-based websites. Sex Educ. 2022;22(3):335-47.
55. Dodge LE, Aguayo R, Dutton C, Hacker MR. The influence of advertising policy on information available online for abortion self-referral. Contraception. 2022;114:61-6.
56. Dong YJ, Zhang LS, Lam C, Huang ZW. Counteracting sexual and reproductive health misperceptions: Investigating the roles of stigma, misinformation exposure, and information overload. Patient Educ Couns. 2024;120:8.
57. D'Souza RS, D'Souza S, Sharpe EE. YouTube as a source of medical information about epidural analgesia for labor pain. Int J Obstet Anesth. 2021;45:133-7.
58. Dubin JM, Aguiar JA, Lin JS, Greenberg DR, Keeter MK, Fantus RJ, et al. The broad reach and inaccuracy of men's health information on social media: analysis of TikTok and Instagram. Int J Impot Res. 2024;36(3):256-60.
59. Duggan J. Using TikTok to teach about abortion: combatting stigma and miseducation in the United States and beyond. Sex Educ. 2023;23(1):81-95.
60. Edinger A, Valdez D, Walsh-Buhi E, Trueblood JS, Lorenzo-Luaces L, Rutter LA, et al. Misinformation and Public Health Messaging in the Early Stages of the Mpox Outbreak: Mapping the Twitter Narrative With Deep Learning. J Med Internet Res. 2023;25:e43841.
61. Ellis SJ. Are women-who-have-sex-with-women an 'at-risk' group for cervical cancer? An exploratory study of women in Aotearoa New Zealand. Sex Health. 2024;21(1):Null.
62. Elmalky O. Restricting sexual and reproductive health rights (SRHR) in Egypt sparks wave of misinformation: SMEX digital rights fund; 2024 [Available from: https://smex.org/restricting-sexual-and-reproductive-health-rights-srhr-in-egypt-sparks-wave-of-disinformation/.
63. Ennab F, Babar MS, Khan AR, Mittal RJ, Nawaz FA, Essar MY, et al. Implications of social media misinformation on COVID-19 vaccine confidence among pregnant women in Africa. Clin Epidemiol Glob Health. 2022;14:100981.
64. Fairchild R, Price M, Craig A, Dotters-Katz SK. Reliability and comprehensiveness of YouTube videos about the COVID-19 vaccine in pregnancy. Am J Infect Control. 2023;51(11):1282-4.
65. Farrugia A, Waling A, Pienaar K, Fraser S. The "Be All and End All"? Young People, Online Sexual Health Information, Science and Skepticism. Qual Health Res. 2021;31(11):2097-110.
66. Faustino G, Silva MOD, Almeida Filho AJ, Ferreira MA. Outline of a project for nursing health education on the Instagram social network. Rev Bras Enferm. 2023;76(2):e20220301.
67. Felix M, Sobel L, Salganicoff A. The Right to Contraception: State and Federal Actions, Misinformation, and the Courts: Keiser Family Foundation, May 23, 2024; 2024 [Available from: https://www.kff.org/womens-health-policy/issue-brief/the-right-to-contraception-state-and-federal-actions-misinformation-and-the-courts/.
68. Fitzgerald MP, Langenderfer J, Renzelli-Cain R, Critch EA. Marketing and Public Policy Implications of Dobbs v. Jackson Women's Health Organization. J Public Policy Mark. 2023;42(4):303-25.
69. Flanders CE, Dinh RN, Pragg L, Dobinson C, Logie CH. Young Sexual Minority Women's Evaluation Processes of Online and Digital Sexual Health Information. Health Commun. 2021;36(10):1286-94.
70. Fleming M, Dehlendorf C, Bell AJ. Misinformation around Birth Control Online Is a Form of Contraception Coercion. Scientific American. 2024.
71. Flew T, Humphry J, Gray J, Hutchinson J, Page Jeffrey C, Johnson M, et al. Submission 154 to the Joint Select Committee on Social Media and Australian Society. 2024.
72. Fode M, Nolsøe AB, Jacobsen FM, Russo GI, Østergren PB, Jensen CFS, et al. Quality of Information in YouTube Videos on Erectile Dysfunction. Sex Med. 2020;8(3):408-13.
73. Foran T. Contraception and the media: lessons past, present and future. Eur J Contracept Reprod Health Care. 2019;24(1):80-2.
74. Foster J, Sarlashkar P, Abraham O, Negris O, Lanthier J, Krapf J, et al. Tiktok as a source of education and misinformation in lichen sclerosus. J Low Genit Tract Dis. 2024:10.1097/LGT.0000000000000846.
75. Frank R. Miss-Conceptions: Abortifacients, Regulatory Failure, and Political Opportunity. The Yale Law Journal. 2019;129(1):208-50.
76. Frid G, Bogaert K, Chen KT. Mobile Health Apps for Pregnant Women: Systematic Search, Evaluation, and Analysis of Features. J Med Internet Res. 2021;23(10):e25667.
77. Galiano V, Orvieto R, Machtinger R, Nahum R, Garzia E, Sulpizio P, et al. "Add-Ons" for Assisted Reproductive Technology: Do Patients Get Honest Information from Fertility Clinics' Websites? Reprod Sci. 2021;28(12):3466-72.
78. Gantt-Shafer J. "They Just Went After Us:" Reproductive Justice Advocacy at an Abortion Fund. Front Commun. 2020;5.
79. Garcia-Iglesias J, Heaphy B, Mowlabocus S, Yodovich N, Nagington M, Patton K, et al. Dating apps as health allies? Examining the opportunities and challenges of dating apps as partners in public health. Med Humanit. 2024;50(3):594-7.
80. Geraets N. ‘So far from science’: GPs desperate to debunk sexual health myths on social media. The Sydney Morning Herald. 2023.
81. Geskin S, Mangaldas L, Philpot A, Modi EK. Battling Misinformation and Disinformation:Sexual and Reproductive Health and Rights in theDigital Age: USC Institute on Inequalities in Global Health; 2024 [Available from: https://globalhealth.usc.edu/2024/02/06/can-reparations-help-to-end-racial-disparities-in-health-2/.
82. Giacometti CF, Galfano GS, Wajman DS, Cordioli E, Beck APA, Podgaec S. Internet use by pregnant women during prenatal care. Einstein (Sao Paulo). 2024;22:eAO0447.
83. Gilbert SF. "When does human life begin?" teaching human embryology in the context of the American abortion debate. Dev Biol. 2024;515:102-11.
84. Glauser W. Abortion clinic websites blocked at Toronto hospital. Cmaj. 2020;192(36):E1049-e50.
85. Golder S, McRobbie-Johnson ACE, Klein A, Polite FG, Gonzalez Hernandez G. Social media and COVID-19 vaccination hesitancy during pregnancy: a mixed methods analysis. Bjog. 2023;130(7):750-8.
86. Good MM, Tanouye S. Social Media Superpowers in Obstetrics and Gynecology. Obstet Gynecol Clin North Am. 2021;48(4):787-800.
87. Gourounti K, Sarantaki A, Dafnou ME, Hadjigeorgiou E, Lykeridou A, Middleton N. A qualitative study of assessing learning needs and digital health literacy in pregnancy: Baby Buddy Forward Greek findings. Eur J Midwifery. 2022;6:55.
88. Green J, Petty J, Whiting L, Orr F, Smart L, Brown AM, et al. 'Blurred boundaries': When nurses and midwives give anti-vaccination advice on Facebook. Nurs Ethics. 2022;29(3):552-68.
89. Grov C, Westmoreland DA, D'Angelo AB, Johnson J, Nash D, Daskalakis DC. Marketing of Tenofovir Disoproxil Fumarate (TDF) Lawsuits and Social Media Misinformation Campaigns' Impact on PrEP Uptake Among Gender and Sexual Minority Individuals. AIDS Behav. 2021;25(5):1396-404.
90. Grünebaum A, Chervenak J, Pollet SL, Katz A, Chervenak FA. The exciting potential for ChatGPT in obstetrics and gynecology. Am J Obstet Gynecol. 2023;228(6):696-705.
91. Gunnarsson L, Wemrell M. Assessing the validity of counter-authority knowledge: the case of Swedish women’s epistemic patchworking around the risks of copper IUD use. J Crit Realism. 2024.
92. Hann LR, Becker A. The option to look: Patient-centred pregnancy tissue viewing at independent abortion clinics in the United States. Sexual and Reproductive Health Matters. 2020;28(1):500-13.
93. Harcourt E, Bering J, Gullam J. Opposition to abortion related to inaccurate beliefs about fetal pain perception in utero. Aust N Z J Obstet Gynaecol. 2021;61(4):599-603.
94. Harlow AF, Willis SK, Smith ML, Rothman EF. Bystander Prevention for Sexual Violence: #HowIWillChange and Gaps in Twitter Discourse. J Interpers Violence. 2021;36(11-12):Np5753-np71.
95. Harris R. Green scarves and data harvesting: How the abortion battle has gone digital. Harvard International Review. 2021;42(2):29-33.
96. Hate CfCD. Profiting from Deceit: How Google Profits From Anti-Choice Ads Distorting Searches For Reproductive Healthcare. 2023.
97. Hayman M, Keppel M, Stanton R, Thwaite TL, Alfrey KL, Alley S, et al. A mixed-methods exploration of attitudes towards pregnant Facebook fitness influencers. BMC Public Health. 2023;23(1):569.
98. Herbenick D, Guerra-Reyes L, Patterson C, Wilson J, Rosenstock Gonzalez YR, Voorheis E, et al. #ChokeMeDaddy: A Content Analysis of Memes Related to Choking/Strangulation During Sex. Arch Sex Behav. 2023;52(3):1299-315.
99. Herbenick D, Patterson C, Khan S, Voorheis E, Sullivan A, Wright P, et al. "Don't Just Randomly Grab Someone's Neck during Intercourse!" An Analysis of Internet Articles about Choking/Strangulation during Sex. J Sex Marital Ther. 2023;49(1):41-55.
100. Hlavinka E. TikTok is being flooded with birth control misinformation. Is it stopping women from taking it? Salon. 2024.
101. Hohmann-Marriott BE, Williams TA, Girling JE. The role of menstrual apps in healthcare: provider and patient perspectives. N Z Med J. 2023;136(1570):42-53.
102. Holly L. Health in the Digital Age: Where Do Children’s Rights Fit In? Health and Human Rights. 2020;22(2):49-54.
103. Hong C. Mpox on Reddit: a Thematic Analysis of Online Posts on Mpox on a Social Media Platform among Key Populations. J Urban Health. 2023;100(6):1264-73.
104. Hu MY, Ho DR, Weinberger JM, Osadchiy V, Mills JN, Eleswarapu SV. Guideline-Nonconformant Investigational Treatments for Erectile Dysfunction: What are Patients Learning? Urology. 2023;173:111-8.
105. International A. Tech and Reproductive Rights 2024 [Available from: https://www.amnestyusa.org/issues/technology/tech-and-repro/.
106. International P. State of the World's Girls 2021: The Truth Gap. Plan International; 2021.
107. Ishida JH, Zhang AJ, Steigerwald S, Cohen BE, Vali M, Keyhani S. Sources of Information and Beliefs About the Health Effects of Marijuana. J Gen Intern Med. 2020;35(1):153-9.
108. Jahnke H, Henrich N, Salem W, Shah N. Evaluating the evidence base for TikTok videos on strategies for becoming pregnant. O&G Open. 2024.
109. John JN, Kabra R, Cappola AR. Counseling Adolescents About Intrauterine Device Insertional Pain in the Social Media Era: Reproductive Justice Principles. J Adolesc Health. 2024;75(6):849-50.
110. Kane G, Harrod J, Vernaelde J, Singh M. Out of Bounds: Foreign and Digital Influence Targeting LGBTI Rights in Ghana. Ipas; 2024.
111. Kawchuk G, Hartvigsen J, Innes S, Simpson JK, Gushaty B. The use of internet analytics by a Canadian provincial chiropractic regulator to monitor, evaluate and remediate misleading claims regarding specific health conditions, pregnancy, and COVID-19. Chiropr Man Therap. 2020;28(1):24.
112. Kedzior SGE, Bianco-Miotto T, Breen J, Diener KR, Donnelley M, Dunning KR, et al. It takes a community to conceive: an analysis of the scope, nature and accuracy of online sources of health information for couples trying to conceive. Reprod Biomed Soc Online. 2019;9:48-63.
113. Khan I. A/78/288: Gendered disinformation and its implications for the right to freedom of expression – Report of the Special Rapporteur on the promotion and protection of the right to freedom of opinion and expression. United Nations; 2023.
114. Khosla R, Mishra V, Singh S. Sexual and reproductive health and rights and bodily autonomy in a digital world. Sex Reprod Health Matters. 2023;31(4):2269003.
115. Kim SJ, Schiffelbein JE, Imset I, Olson AL. Countering Antivax Misinformation via Social Media: Message-Testing Randomized Experiment for Human Papillomavirus Vaccination Uptake. J Med Internet Res. 2022;24(11):e37559.
116. Kirana PS, Gudeloglu A, Sansone A, Fode M, Reisman Y, Corona G, et al. E-Sexual Health: A Position Statement of the European Society for Sexual Medicine. J Sex Med. 2020;17(7):1246-53.
117. Kohler RE, Hemler J, Wagner RB, Sullivan B, Macenat M, Tagai EK, et al. Confusion and anxiety in between abnormal cervical cancer screening results and colposcopy: "The land of the unknown". Patient Educ Couns. 2023;114:107810.
118. Laestadius LI, Van Hoorn K, Wahl M, Witt A, Carlyle KE, Guidry JPD. Promotion of an Algorithm-Based Tool for Pregnancy Prevention by Instagram Influencers. J Womens Health (Larchmt). 2024;33(2):141-51.
119. Lahti H, Kokkonen M, Hietajärvi L, Lyyra N, Paakkari L. Social media threats and health among adolescents: evidence from the health behaviour in school-aged children study. Child Adolesc Psychiatry Ment Health. 2024;18(1):62.
120. Lamačková A, Yoshida K, Gervais C, Hoctor L, Mirahver L, Thomasen K. Sexual and reproductive health and rights in Europe: Progress and challenges - 2024 Follow-up report to the 2017 Issue Paper by the Council of Europe Commissioner for Human Rights. Council of Europe; 2024.
121. Lang JJ, Giffen Z, Hong S, Demeter J, El-Zawahry A, Sindhwani P, et al. Assessing Vasectomy-Related Information on YouTube: An Analysis of the Quality, Understandability, and Actionability of Information. Am J Mens Health. 2022;16(2):15579883221094716.
122. Lee KN, Joo YJ, Choi SY, Park ST, Lee KY, Kim Y, et al. Content Analysis and Quality Evaluation of Cesarean Delivery-Related Videos on YouTube: Cross-sectional Study. J Med Internet Res. 2021;23(7):e24994.
123. Lee SY, Lee S. Quality of Online Information Regarding High-Risk Pregnancies. Comput Inform Nurs. 2021;39(12):974-83.
124. Levy MS, Hunt KN, Rinehart S, Brown AD, Kelly AG, Sundaram P, et al. COVID-19 Vaccine Information and Infertility Posts on X: Insights on a Misinformation Pandemic. Perm J. 2024;28(2):47-54.
125. Li MY, Li GF, Yang SJ. Correction by distraction: how high-tempo music enhances medical experts' debunking TikTok videos. J Comput-Mediat Commun. 2024;29(5):15.
126. Liang OS, Chen Y, Bennett DS, Yang CC. Identifying Self-Management Support Needs for Pregnant Women With Opioid Misuse in Online Health Communities: Mixed Methods Analysis of Web Posts. J Med Internet Res. 2021;23(2):e18296.
127. Libretti A, Vitale SG, Saponara S, Corsini C, Aquino CI, Savasta F, et al. Hysteroscopy in the new media: quality and reliability analysis of hysteroscopy procedures on YouTube™. Arch Gynecol Obstet. 2023;308(5):1515-24.
128. Loeb S, Sanchez Nolasco T, Byrne N, Allen L, Langford AT, Ravenell JE, et al. Qualitative Study on Internet Use and Care Impact for Black Men With Prostate Cancer. Health Educ Behav. 2024;51(3):359-66.
129. London CUo. TikTok and Instagram are full of misleading information about birth control — and wellness influencers are helping drive these narratives 2024 [Available from: https://www.city.ac.uk/news-and-events/news/2024/april/social-media-birth-control-misinformation.
130. Looi M-K. The grey area of “fertility tech” being mis-sold as contraception. bmj. 2024;384.
131. Lovett J, Gordon C, Patton S, Chen CX. Online information on dysmenorrhoea: An evaluation of readability, credibility, quality and usability. J Clin Nurs. 2019;28(19-20):3590-8.
132. Madung O. Exporting disinformation: How foreign groups peddle influence in Kenya through Twitter. Mozilla Foundation; 2023.
133. Malcolm HE. Pregnancy centers and the limits of mandated disclosure. Columbia Law Review. 2019;119(4):1133-68.
134. Malhotra K, Kempegowda P. Appraising Unmet Needs and Misinformation Spread About Polycystic Ovary Syndrome in 85,872 YouTube Comments Over 12 Years: Big Data Infodemiology Study. J Med Internet Res. 2023;25:e49220.
135. Malki LM, Patel D, Singh A, Abdelnour Nocera J, Kristín Lárusdóttir M, Petrie H, et al., editors. A Mixed-Methods Analysis of Women’s Health Misinformation on Social Media. Lect Notes Comput Sci; 2023: Springer Science and Business Media Deutschland GmbH.
136. Malki LM, Patel D, Singh A, editors. A Mixed-Methods Analysis of Women's Health Misinformation on Social Media. 19th International-Federation-for-Information-Processing-Technical-Committee- 13 (IFIP TC13) International Conference on Human-Computer Interaction (INTERACT); 2023 Aug 28-Sep 01; Univ York, York, ENGLAND. CHAM: Springer International Publishing Ag; 2023.
137. Marrinan C. Geofencing: The Overlooked Barrier to Reproductive Freedom: Council on Foreign Relations; 2024 [Available from: https://www.cfr.org/blog/geofencing-overlooked-barrier-reproductive-freedom.
138. Martin S, Kilich E, Dada S, Kummervold PE, Denny C, Paterson P, et al. "Vaccines for pregnant women…?! Absurd" - Mapping maternal vaccination discourse and stance on social media over six months. Vaccine. 2020;38(42):6627-37.
139. Martinez A. The Dangers of birth control misinformation Post-Roe. Think Global Health. 2024.
140. Martins-Filho PR. Increase in interest in sexually transmitted infections on YouTube during the monkeypox outbreak in 2022: A global infodemiology study. Int J Surg. 2022;107.
141. Matos C. Community engagement with health messages on reproductive health in an age of misinformation and political polarisation: A case study of the US NGO open arms in Florida 1 , 2. Young Adulthood Across Digital Platforms: Emerald Publishing Limited; 2024. p. 145-64.
142. Matos C. Making sense of reproductive health messages in the Global South: A case study of Brazil’s NGO Reprolatina. Int J Media Cult Polit. 2023;19(2-3):237-54.
143. McCammon HJ. A War of Words Over Abortion: The Legal-Framing Contest Over the Undue Burden Standard. The Justice System Journal. 2022;43(4):623-44.
144. McCarthy H, Others. How Mpox anti-vaxx conspiracies target and stigmatise LGBTQ+ people. 2024.
145. McCarthy R, Byrne G, Brettle A, Choucri L, Ormandy P, Chatwin J. Midwife-moderated social media groups as a validated information source for women during pregnancy. Midwifery. 2020;88:102710.
146. McInroy LB, McCloskey RJ, Craig SL, Eaton AD. LGBTQ+ Youths' Community Engagement and Resource Seeking Online versus Offline. J Technol Human Serv. 2019;37(4):315-33.
147. McMahon E, Fetters T, Jive NL, Mpoyi M. Perils and promise providing information on sexual and reproductive health via the Nurse Nisa WhatsApp chatbot in the Democratic Republic of the Congo. Sexual and Reproductive Health Matters. 2023;31(4).
148. McMahon HV, McMahon BD. Automating untruths: ChatGPT, self-managed medication abortion, and the threat of misinformation in a post-Roe world. Front Digit Health. 2024;6:1287186.
149. Miller NS. How influencers and content creators discuss birth control on social media: What research shows. The Journalist’s Resource. 2024.
150. Mom C. INSIGHT: How misinformation about Samoa Agreement caused uproar in Nigeria: African Digital Democracy Observatory; 2024 [Available from: https://disinfo.africa/insight-how-misinformation-about-samoa-agreement-caused-uproar-in-nigeria-c85c6f079752.
151. Morra S, Napolitano L, Collà Ruvolo C, Celentano G, La Rocca R, Capece M, et al. Could YouTubeTM encourage men on prostate checks? A contemporary analysis. Arch Ital Urol Androl. 2022;94(3):285-90.
152. Movahedi Nia Z, Bragazzi N, Asgary A, Orbinski J, Wu J, Kong J. Mpox Panic, Infodemic, and Stigmatization of the Two-Spirit, Lesbian, Gay, Bisexual, Transgender, Queer or Questioning, Intersex, Asexual Community: Geospatial Analysis, Topic Modeling, and Sentiment Analysis of a Large, Multilingual Social Media Database. J Med Internet Res. 2023;25:e45108.
153. Murciano-Gamborino C, Diez-Domingo J, Fons-Martinez J. Healthcare Professionals' Perspectives on HPV Recommendations: Themes of Interest to Different Population Groups and Strategies for Approaching Them. Vaccines (Basel). 2024;12(7).
154. Myles D. Grindr? it's a "Blackmailer's goldmine"! The weaponization of queer data publics Amid the US-China trade conflict. Sexualities. 2024;27(7):1205-24.
155. Neigh S. Podcast: Challenging mis- and disinformation about sexual health and rights: Talking Radical; 2023 [Available from: https://talkingradical.ca/2023/02/07/radio-challenging-mis-and-disinformation-about-sexual-health-and-rights/?utm_source=chatgpt.com.
156. Newns KVJ, Feldman MD. Munchausen by internet and false perinatal crises. Ann Clin Psychiatry. 2021;33(2):e8-e12.
157. Nicolla S, Lazard AJ. Social Media Communication About Sexual Violence May Backfire: Online Experiment with Young Men. J Health Commun. 2023;28(1):28-37.
158. Nimbi FM, Ricciardi L, Galizia R, Lingiardi V. Using social media for male sexual health: what do we miss? A perspective article on good practices and missing evidence. Int J Impot Res. 2024;36(7):679-83.
159. Nörby U, Noël-Cuppers B, Hristoskova S, Desai M, Härmark L, Steel M, et al. Online information discrepancies regarding safety of medicine use during pregnancy and lactation: an IMI ConcePTION study. Expert Opin Drug Saf. 2021;20(9):1117-24.
160. Norris S. Kenya’s Pro-Choice Movement Faces Emboldened Threats in a Post-Roe World. Byline Times. 2022.
161. Obstacles to autonomy: Post-Roe removal of abortion information online. Amnesty International; 2024.
162. Olamijuwon E, Clifford O, Adjiwanou V. Understanding how young African adults interact with peer-generated sexual health information on Facebook and uncovering strategies for successful organic engagement. BMC Public Health. 2021;21(1):2153.
163. Onder CE, Koc G, Gokbulut P, Taskaldiran I, Kuskonmaz SM. Evaluation of the reliability and readability of ChatGPT-4 responses regarding hypothyroidism during pregnancy. Sci Rep. 2024;14(1):243.
164. Ontiveros ST, Qozi M, Cantrell FL. A Concerning Internet Trend That Might Result in Unintended Pregnancy. Am J Med. 2020;133(8):e427.
165. Organization WH. The role of artificial intelligence in sexual and reproductive health and rights. 2024.
166. Pagoto SL, Palmer L, Horwitz-Willis N. The Next Infodemic: Abortion Misinformation. J Med Internet Res. 2023;25:e42582.
167. Pandian T, Maraimalai N. Understanding cybercrime's impact on women's physical and psychological well-being. Afr J Reprod Health. 2024;28(5):103-12.
168. Pang RD, Dormanesh A, Hoang Y, Chu M, Allem JP. Twitter Posts About Cannabis Use During Pregnancy and Postpartum:A Content Analysis. Subst Use Misuse. 2021;56(7):1074-7.
169. Park HJ. The rise of generative artificial intelligence and the threat of fake news and disinformation online: Perspectives from sexual medicine. Investig Clin Urol. 2024;65(3):199-201.
170. Pecoriello J, Yoder N, Smith MB, Blakemore JK. Keeping you posted: analysis of fertility-related social media posts after introduction of the COVID-19 vaccine. Eur J Contracept Reprod Health Care. 2023;28(3):168-72.
171. Pereira CG, Santos RF, Faria APV, Silva T, Pena É D, Matozinhos FP. Reliability of information available on popular websites about vaccination of pregnant women. Rev Esc Enferm USP. 2021;55:e20200517.
172. Pereira-Kotze C, Horwood C, Haskins L, Kingston G, Luthuli S, Doherty T. Exploring women's exposure to marketing of commercial formula products: a qualitative marketing study from two sites in South Africa. Glob Health Action. 2022;15(1):2074663.
173. Perez A, Panagiotopoulou E, Curtis P, Roberts R. Barriers and facilitators to mood and confidence in pregnancy and early parenthood during COVID-19 in the UK: mixed-methods synthesis survey. BJPsych Open. 2021;7(4):e107.
174. Pfender E. Social media misinformation is scaring women about birth control: STAT news; 2024 [Available from: https://www.statnews.com/2024/11/05/social-media-misinformation-is-scaring-women-about-birth-control/.
175. Pfender EJ, Caplan SE. The Effect of Social Media Influencer Warranting Cues on Intentions to Use Non-Hormonal Contraception. Health Commun. 2024:1-15.
176. Pfender EJ, Devlin MM. What Do Social Media Influencers Say About Birth Control? A Content Analysis of YouTube Vlogs About Birth Control. Health Commun. 2023;38(14):3336-45.
177. Pfender EJ, Fowler LR. Social Media Is Influencing Contraceptive Choice. J Womens Health (Larchmt). 2024;33(5):563-4.
178. Pfender EJ, Kuijpers KL, Wanzer CV, Bleakley A. Cycle Syncing and TikTok's Digital Landscape: A Reasoned Action Elicitation Through a Critical Feminist Lens. Qual Health Res. 2024:10497323241297683.
179. Pizzarossa LB, Nandagiri R. Self-managed abortion: a constellation of actors, a cacophony of laws? Sexual and Reproductive Health Matters. 2021;29(1):23-30.
180. Pleasants E, Guendelman S, Weidert K, Prata N. Quality of top webpages providing abortion pill information for Google searches in the USA: An evidence-based webpage quality assessment. PLoS One. 2021;16(1):e0240664.
181. Pleasure ZH, Becker A, Johnson DM, Broussard K, Lindberg L. How TikTok is being used to talk about abortion post-Roe: A content analysis of the most liked abortion TikToks. Contraception. 2024;133:110384.
182. Programme UND. Information Asymmetries in the Digital Sexual and Reproductive Health Space. United Nations Development Programme; 2021.
183. Ranney ML, Gostin LO. State Medical Board Sanctions for Misinformation Should Be Rare. JAMA Netw Open. 2024;7(11):e2443878.
184. Rasouli MA, Sagun BK, Verma K, Duke CM. Black infertility and social media engagement: a mixed methodology analysis. F S Rep. 2022;3(2 Suppl):55-61.
185. Reproaction. Reproductive Rights and Justice Movement Leaders Respond to ‘Big Tech’ Suppression of Accurate Abortion Information. 2023.
186. ReproBlueprint. Blueprint for Sexual and Reproductive Health, Rights, and Justice Policy Agenda. 2023
187. Restrepo NJ, Illari L, Leahy R, Sear RF, Lupu Y, Johnson NF. How Social Media Machinery Pulled Mainstream Parenting Communities Closer to Extremes and Their Misinformation During Covid-19. IEEE Access. 2022;10:2330-44.
188. Rielly B, Betteridge-Moes M, Misikir M. Fertile Ground - The Rising Anti-Abortion Movement in Ethiopia: A Threat to Women's Rights. New Internationalist. 2024.
189. Rights ACfSH. Government Takes Action to Combat Anti-choice Misinformation. Action Canada for Sexual Health & Rights; 2024.
190. Rights CfR. Submission to OHCHR Report on freedom of expression and the gender dimensions of disinformation. Center for Reproductive Rights; 2023.
191. Şahin MF, Keleş A, Özcan R, Doğan Ç, Topkaç EC, Akgül M, et al. Evaluation of information accuracy and clarity: ChatGPT responses to the most frequently asked questions about premature ejaculation. Sex Med. 2024;12(3):qfae036.
192. SAHM-NASPAG. Crisis Pregnancy Centers in the U.S.: Lack of Adherence to Medical and Ethical Practice Standards: A Joint Position Statement of the Society for Adolescent Health and Medicine and the North American Society for Pediatric and Adolescent Gynecology. J Adolesc Health. 2019;65(6):821-4.
193. Sajjadi NB, Nowlin W, Nowlin R, Wenger D, Beal JM, Vassar M, et al. United States internet searches for "infertility" following COVID-19 vaccine misinformation. J Osteopath Med. 2021;121(6):583-7.
194. Sandset T, Pham T, Dong DD, Davis SLM. Sexual and reproductive health and digital human rights: a study with people living with HIV and key populations in Vietnam. Cult Health Sex. 2024:1-16.
195. Schmall E, Maheshwari S. Abortion Groups Say Tech Companies Suppress Posts and Accounts. The New York Times. 2024.
196. Security JCfH. Understanding common rumors that emerge during public health emergencies 2023 [Available from: https://centerforhealthsecurity.org/our-work/research-projects/trust-in-public-health/tackling-rumors/trust-common-rumors-during-phes.
197. Šepec M, Lango M. Virtual revenge pornography as a new online threat to sexual integrity. Balkan Soc Sci Rev. 2020;15(15):117-34.
198. Serçekuş P, Değirmenciler B, Özkan S. Internet use by pregnant women seeking childbirth information. J Gynecol Obstet Hum Reprod. 2021;50(8):102144.
199. Shackleford M, Horvath A, Repetto M, Thi A, Twells R, Sanders M, et al. An analysis of oral contraceptive related videos on TikTok. AJOG Glob Rep. 2024;4(3):100364.
200. Shao YH, Tulandi T, Abenhaim HA. Evaluating the Quality and Reliability of Online Information on Social Fertility Preservation. J Obstet Gynaecol Can. 2020;42(5):561-7.
201. Shay LA, McKenzie A, Avshman E, Savas LS, Shegog R. HPV vaccine-related articles shared on Facebook from 2019 to 2021: Did COVID make a difference? PEC Innov. 2024;4.
202. Sherman J. How Abortion Misinformation and Disinformation Spread Online: Scientific American; 2022 [Available from: https://www.scientificamerican.com/article/how-abortion-misinformation-and-disinformation-spread-online/.
203. Shoureshi PS, Rajasegaran A, Kokorowski P, Sparks SS, Seideman CA. Social media engagement, perpetuating selected information, and accuracy regarding CA SB-201: Treatment or intervention on the sex characteristics of a minor. J Pediatr Urol. 2021;17(3):372-7.
204. Sidnell A, Nestel P. UK Internet antenatal dietary advice: a content accuracy and readability analysis. Br J Nutr. 2020;124(10):1061-8.
205. Silva M, Anaba U, Jani Tulsani N, Sripad P, Walker J, Aisiri A. Gender-Based Violence Narratives in Internet-Based Conversations in Nigeria: Social Listening Study. J Med Internet Res. 2023;25:e46814.
206. Simas C, Larson HJ, Paterson P. "Saint Google, now we have information!": a qualitative study on narratives of trust and attitudes towards maternal vaccination in Mexico City and Toluca. BMC Public Health. 2021;21(1):1170.
207. Simmons C, Martiny C. Networks of Dissuasion: Mapping Online Attacks on Reproductive Rights in France. Institute for Strategic Dialogue; 2024.
208. Smith AM, Mucedola AS, Ausness-Ayres A. My Video, My Choice: A Quantitative Content Analysis of U.S. OBGYN TikTok Videos in the Post-Roe Era. Women's Reprod Health. 2024.
209. Snyder K, Pelster AK, Dinkel D. Healthy eating and physical activity among breastfeeding women: the role of misinformation. BMC Pregnancy Childbirth. 2020;20(1):470.
210. Social Media Toolkit: Action Canada for Sexual Health and Rights; 2024 [Available from: https://www.actioncanadashr.org/sexual-health-hub/social-media-toolkit.
211. Society CfI. Event Report: Consultation on Gendered Information Disorder in India 2024 [Available from: https://cis-india.org/internet-governance/event-report-consultation-on-gendered-information-disorder-in-india-pdf.
212. Sormunen T, Niklasson B, Westerbotn M. Information Regarding Male Infertility in the Nordic Countries' Fertility Clinics' Websites: An Evaluation of Readability, Suitability, and Quality. J Consum Health Internet. 2024:15.
213. Southerton C, Clark M. OBGYNs of TikTok and the role of misinformation in diffractive knowledge production. J Sociol. 2023;59(3):610-27.
214. Stephan AP, Hauc SC, Marks VA, Bercik R, Rickey L. TikTok misinformation and user engagement in female pelvic floor conditions. Neurourol Urodyn. 2024;43(8):1956-61.
215. Strzyżyńska W. Meta and Google accused of restricting reproductive health information: The Guardian; 2024 [Available from: https://www.theguardian.com/global-development/2024/mar/27/meta-and-google-accused-of-restricting-reproductive-health-information.
216. Sundstrom B, Cartmell KB, White AA, Russo N, Well H, Pierce JY, et al. HPV Vaccination Champions: Evaluating a Technology-Mediated Intervention for Parents. Front Digit Health. 2021;3:636161.
217. Sundstrom B, Cartmell KB, White AA, Well H, Pierce JY, Brandt HM. Correcting HPV vaccination misinformation online: Evaluating the HPV vaccination NOW social media campaign. Vaccines. 2021;9(4).
218. Swartz JJ, Rowe C, Morse JE, Bryant AG, Stuart GS. Women's knowledge of their state's abortion regulations. A national survey. Contraception. 2020;102(5):318-26.
219. Swartzendruber A, English A, Greenberg KB, Murray PJ, Freeman M, Upadhya K, et al. Crisis Pregnancy Centers in the United States: Lack of Adherence to Medical and Ethical Practice Standards; A Joint Position Statement of the Society for Adolescent Health and Medicine and the North American Society for Pediatric and Adolescent Gynecology. J Pediatr Adolesc Gynecol. 2019;32(6):563-6.
220. Sweeney L, Carroll K, Noble-Carr D, Waldby C. Lactation after infant death: an analysis of Australian healthcare agencies' online health information. Health Sociol Rev. 2020;29(1):45-61.
221. Szwarc L, Sánchez-Antelo V, Paolino M, Arrossi S. “You are always left with doubts”: information access among HPV-positive women in Greater Buenos Aires. Rev Salud Publica. 2023;25(2):1-7.
222. Takenaka BP, Kirklewski SJ, Griffith FJ, Gibbs JJ, Lauckner CK, Nicholson E, et al. "It's another gay disease": an intersectional qualitative approach contextualizing the lived experiences of young gay, bisexual, and other sexual minoritized men in the United States during the mpox outbreak. BMC Public Health. 2024;24(1):1574.
223. Topper PS, Bauermeister JA, Golinkoff J. Fertility health information seeking among sexual minority women. Fertil Steril. 2022;117(2):399-407.
224. Truong MD, Abbott JA. #thehorsehasbolted #canwecatchit? #isitworthit? J Minimally Invasive Gynecol. 2021;28(10):1687-8.
225. Unfried K, Priebe J. Who shares fake news on social media? Evidence from vaccines and infertility claims in sub-Saharan Africa. PLoS One. 2024;19(4):e0301818.
226. UNICEF. Digital misinformation/disinformation and children. 2021.
227. UNICEF. Looking out for adolescents and youth from key populations: Formative assessment on the needs of adolescents and youth at risk of HIV: Case studies from Indonesia, the Philippines, Thailand and Viet Nam. UNICEF; 2019.
228. UNICEF. Young people call for increased recognition of their sexual and reproductive health rights 2024 [Available from: https://www.unicef.org/southafrica/stories/young-people-call-increased-recognition-their-sexual-and-reproductive-health-rights.
229. Vågenes H, Pranić SM. Analysis of the quality, accuracy, and readability of patient information on polycystic ovarian syndrome (PCOS) on the internet available in English: a cross-sectional study. Reprod Biol Endocrinol. 2023;21(1):44.
230. Valente PK, Morin C, Roy M, Mercier A, Atlani-Duault L. Sexual transmission of Zika virus on Twitter: A depoliticised epidemic. Glob Public Health. 2020;15(11):1689-701.
231. van Dijk MR, van der Marel AF, van Rheenen-Flach LE, Ganzevoort W, Moll E, Scheele F, et al. YouTube as a Source of Patient Information on External Cephalic Version: Cross-Sectional Study. JMIR Form Res. 2024;8:e50087.
232. Vazquez Corona M, Betrán AP, Bohren MA. The portrayal and perceptions of cesarean section in Mexican media Facebook pages: a mixed-methods study. Reprod Health. 2022;19(1):49.
233. Verma K, Sagun BK, Rasouli MA, Duke CM. Social media engagement with transgender fertility content. F S Rep. 2022;3(2 Suppl):100-5.
234. Vicente-Neira A, Prieto-Gómez V, Navarro-Brazález B, Lirio-Romero C, Bailón-Cerezo J, Torres-Lacomba M. Online Information on Painful Sexual Dysfunction in Women: Quality Analysis of Websites in SPANISH about Dyspareunia, Vaginismus and Vulvodynia. Int J Environ Res Public Health. 2022;19(3).
235. Viera A, van den Berg JJ, Sosnowy CD, Mehta NA, Edelman EJ, Kershaw T, et al. Barriers and Facilitators to HIV Pre-Exposure Prophylaxis Uptake Among Men Who have Sex with Men Who Use Stimulants: A Qualitative Study. AIDS Behav. 2022;26(9):3016-28.
236. Voets A, Wang B, Maslen C, Orza L, Chawla R, Birgin R. Advancing the sexual and reproductive health and rights of women who use drugs: Frontline AIDS; 2020 [Available from: https://frontlineaids.org/wp-content/uploads/2020/02/Guide-for-harm-reduction-programmes-FINAL-24Feb-WEB.pdf.
237. Vogels-Broeke M, Daemers D, Budé L, de Vries R, Nieuwenhuijze M. Sources of information used by women during pregnancy and the perceived quality. BMC Pregnancy Childbirth. 2022;22(1):109.
238. Wahab MSA, Abd Hamid NN, Yassen AO, Naim MJ, Ahamad J, Zulkifli NW, et al. How Internet Websites Portray Herbal Vitality Products Containing Eurycoma longifolia Jack: An Evaluation of the Quality and Risks of Online Information. Int J Environ Res Public Health. 2022;19(19).
239. Wang Y, O'Connor K, Flores I, Berdahl CT, Urbanowicz RJ, Stevens R, et al. Health activism, vaccine, and mpox discourse: BERTopic based mixed-method analyses of tweets from sexual minority men and gender diverse (SMMGD) individuals in the U.S. medRxiv. 2024.
240. Waser M, Heiss R, Borena W. Factors affecting children's HPV vaccination in Austria: Evidence from a parent survey. Hum Vaccin Immunother. 2022;18(6):2126251.
241. Washington I, Yilma H. How Abortion Misinformation Gives Rise to Restrictive Abortion Laws. KFF Health Misinformation Monitor [Internet]. 2024; Volume 9, October 24, 2024. Available from: https://www.kff.org/health-misinformation-monitor/how-abortion-misinformation-gives-rise-to-restrictive-abortion-laws/.
242. Web" Wo, C" P. Bings’ Typo-Searching for Abortion 2024 [Available from: https://repro-uncensored.squarespace.com/research/bing.
243. Weber L, Malhi S. Women are getting off birth control amid misinformation explosion: Washington Post; 2024 [Available from: https://www.washingtonpost.com/health/2024/03/21/stopping-birth-control-misinformation/.
244. Weckend EF, Chandra P. Viral transmissions: Memes as strategies for destigmatizing taboos around sexual health on TikTok. Designing Interactive Systems Conference; 2024/7. New York, NY, USA: ACM; 2024.
245. Wexler A, Davoudi A, Weissenbacher D, Choi R, O'Connor K, Cummings H, et al. Pregnancy and health in the age of the Internet: A content analysis of online "birth club" forums. PLoS One. 2020;15(4):e0230947.
246. Willburger B, Chen Z, Mansfield KJ. Investigation of the quality and health literacy demand of online information on pelvic floor exercises to reduce stress urinary incontinence. Aust N Z J Obstet Gynaecol. 2024.
247. Wilson-Lowe RV, Purcell C, Lewis R, McDaid L. Seeking support for abortion online: A qualitative study of women's experiences. BMJ Sex Reprod Health. 2024;50(3):172-7.
248. Wu J, Greene M, Happ M, Trahair E, Montoya M, Swartz JJ. Medication abortion on TikTok: misinformation or reliable resource? Am J Obstet Gynecol. 2023;228(6):749-51.
249. Yagiz B, Coskun BN, Halil EY, Dalkilic E, Pehlivan Y. The efficacy and reliability of English YouTube videos as a source of information for pregnant rheumatoid arthritis patients. Clin Rheumatol. 2023;42(12):3311-20.
250. Yuksel B, Cakmak K. Healthcare information on YouTube: Pregnancy and COVID-19. Int J Gynaecol Obstet. 2020;150(2):189-93.
251. Zaila KE, Osadchiy V, Shahinyan RH, Mills JN, Eleswarapu SV. Social Media Sensationalism in the Male Infertility Space: A Mixed Methodology Analysis. World J Mens Health. 2020;38(4):591-8.
252. Zemyarska MS. Is it ethical to provide IVF add-ons when there is no evidence of a benefit if the patient requests it? Journal of Medical Ethics. 2019;45(5):346-50.
253. Zhang L, Dong Y, Lam C, Huang Z. Engaging and (the Illusion of) Learning? Examining the Relationship Between Different Social Media Activities and Reproductive Health Knowledge. J Health Commun. 2024;29(5):327-39.
254. Zhang L, Liu PL, Lam C, Huang Z. Well-informed or misinformed? News-Finds-Me perception in shaping sexual and reproductive health knowledge and behavioral intentions among Chinese women. Soc Sci Med. 2024;359:117271.
