# Supplementary material for: Impacts of Sexual and Reproductive Health and Rights Misinformation in Digital Spaces on Human Rights Protection and Promotion: Scoping Review
Source: JMIR Infodemiology. 2025 Dec 30;5:e83747. doi: 10.2196/83747 (PMC12811040; doi:10.2196/83747)
Supplement: Multimedia Appendix 4 [file infodemiology_v5i1e83747_app4.docx]

**Figure S1.** Number of papers and documents by publication year and type.


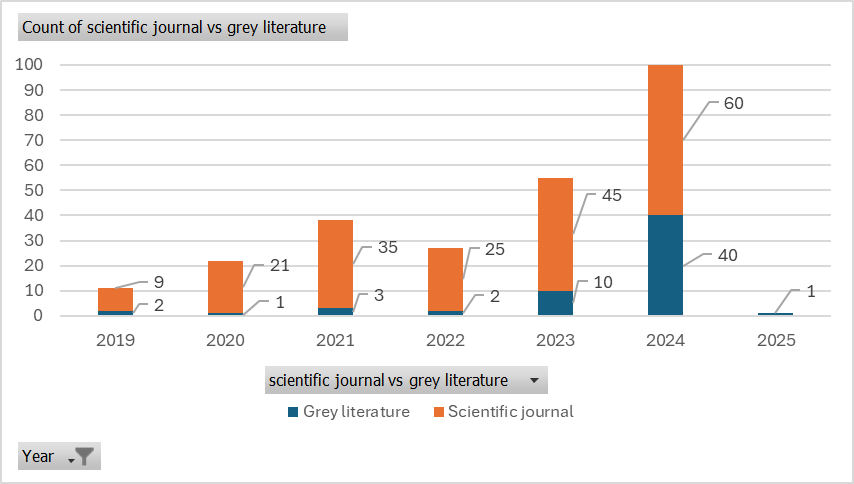


**Figure S2.** Number of times a country was covered by a study/document (if geographical analysis was done).


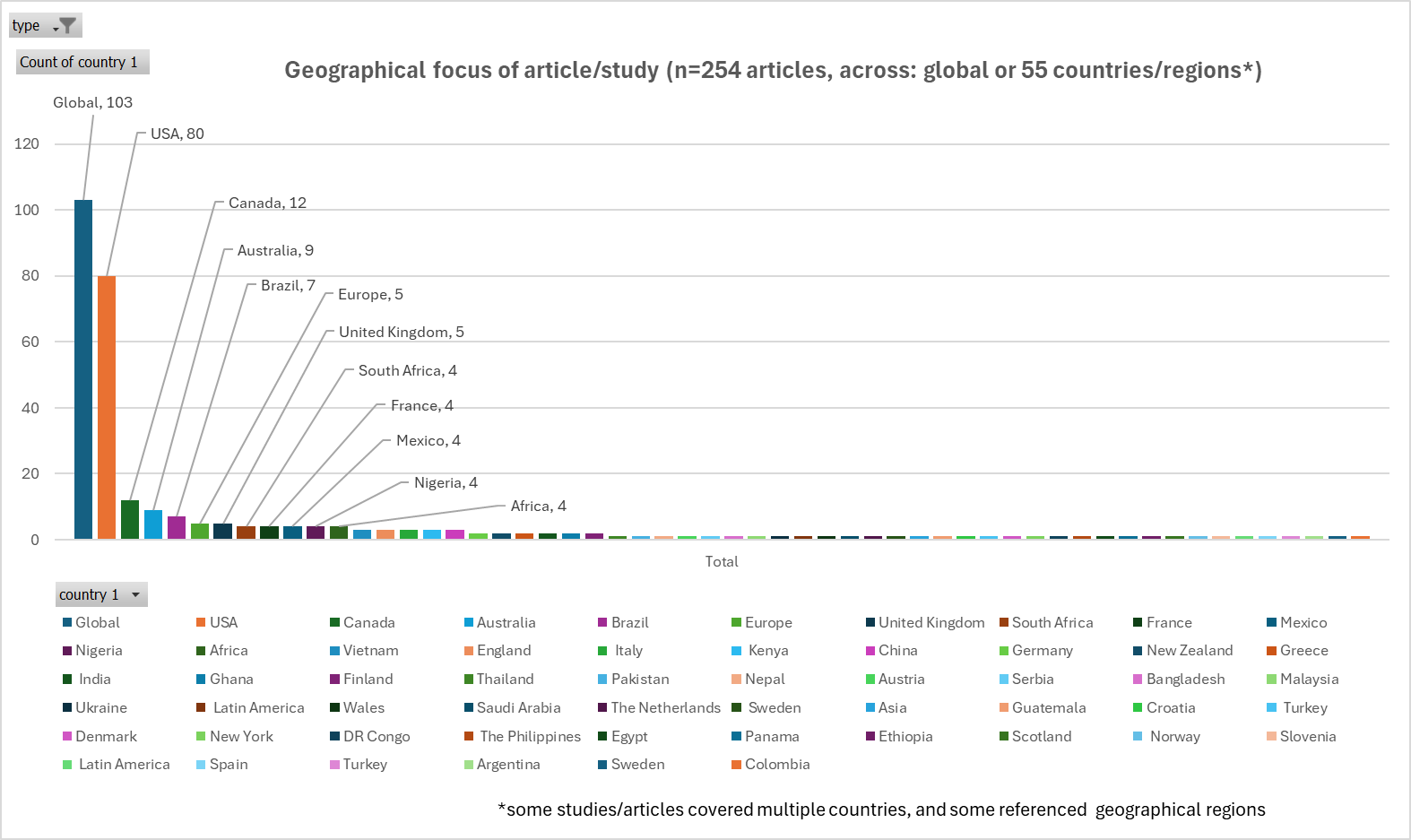


**Figure S3.**


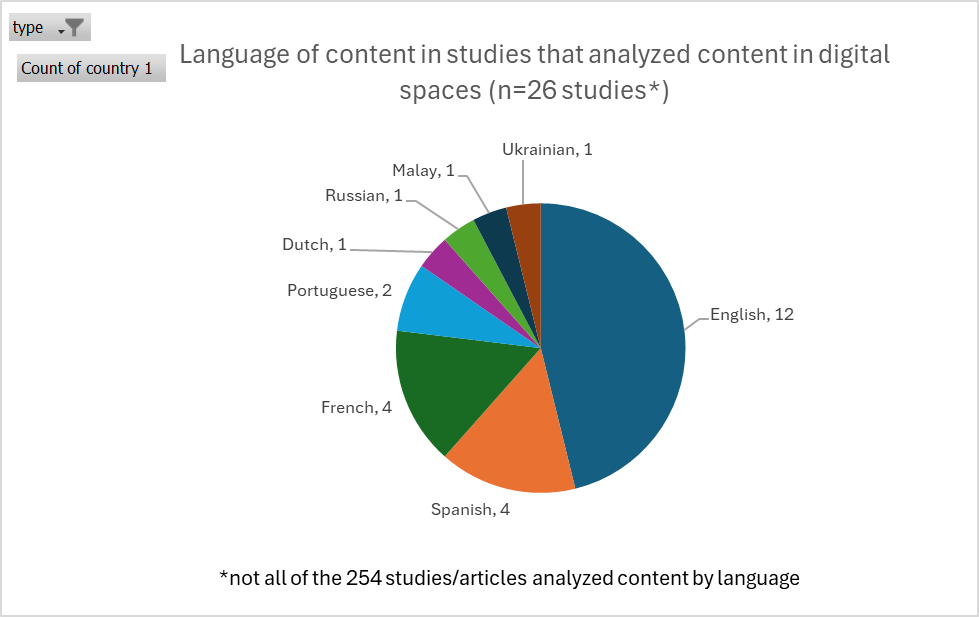


**Figure S4.**


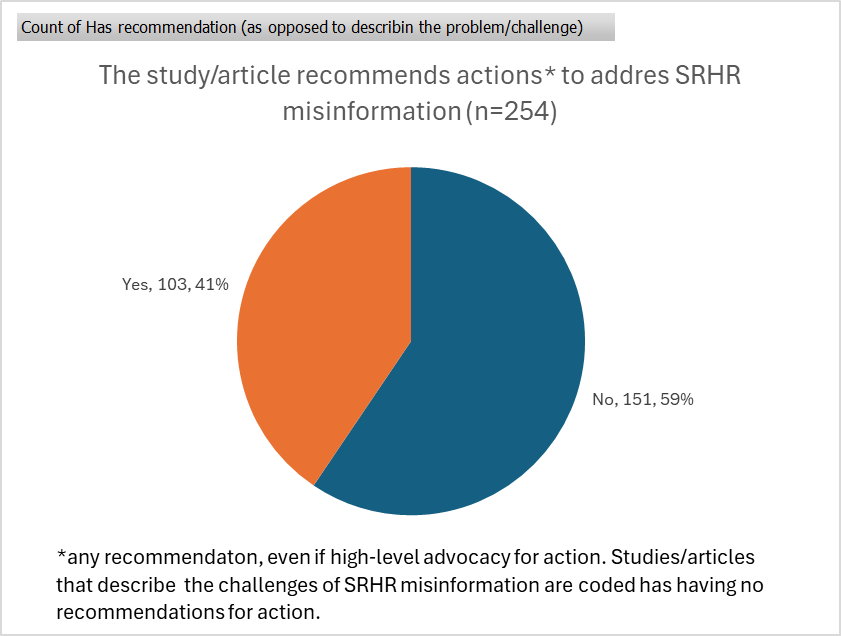


**Figure
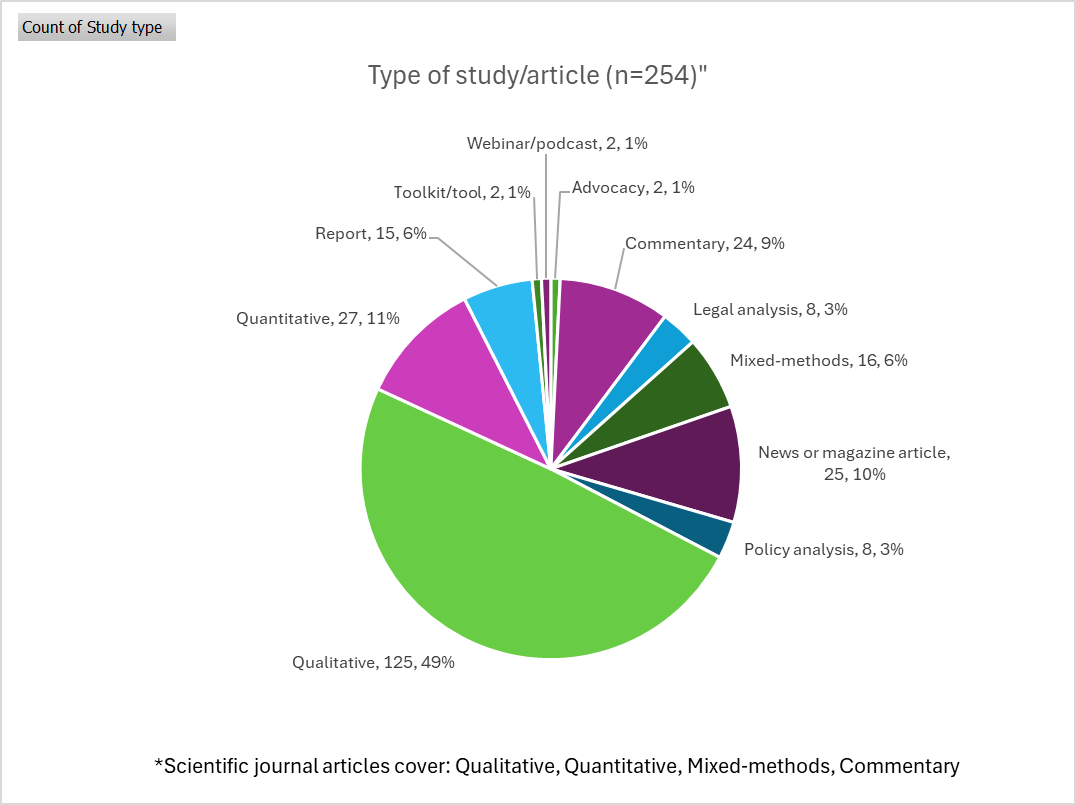
S5.**

**Figure S6.**


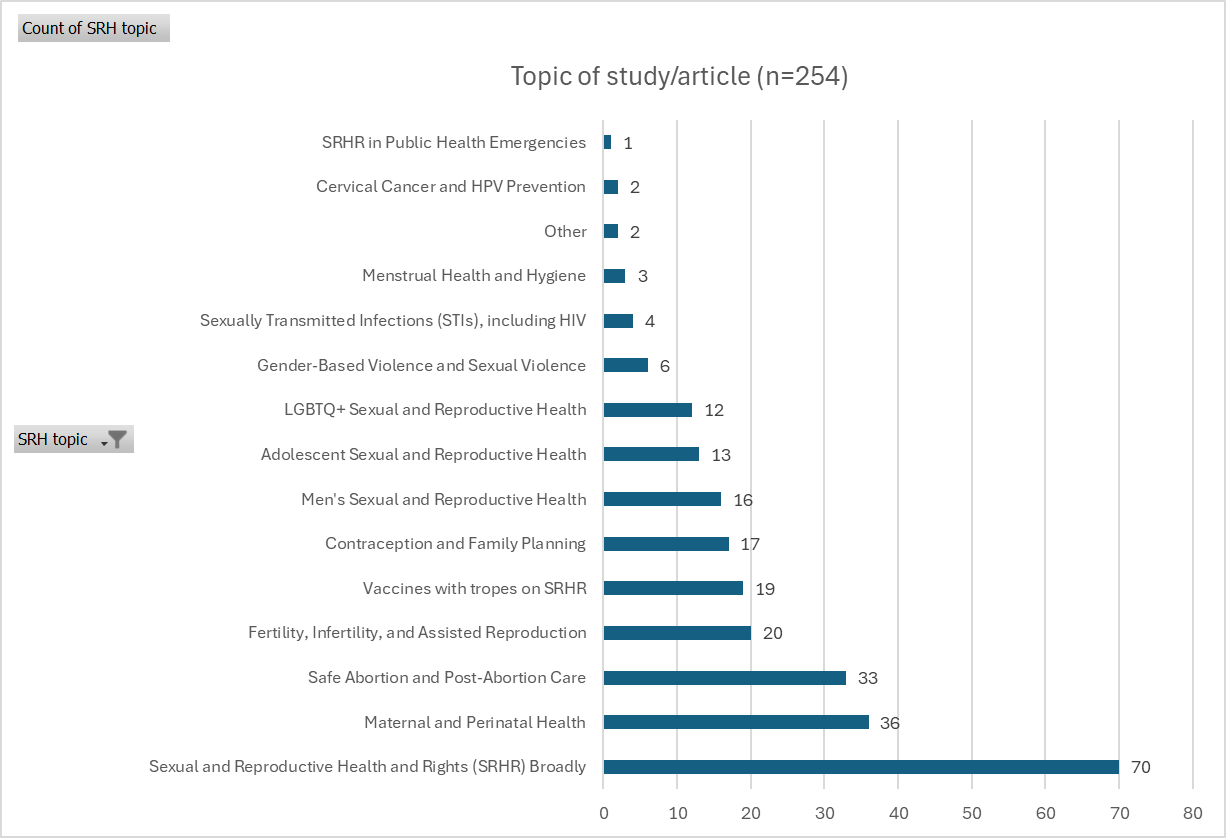


**Figure
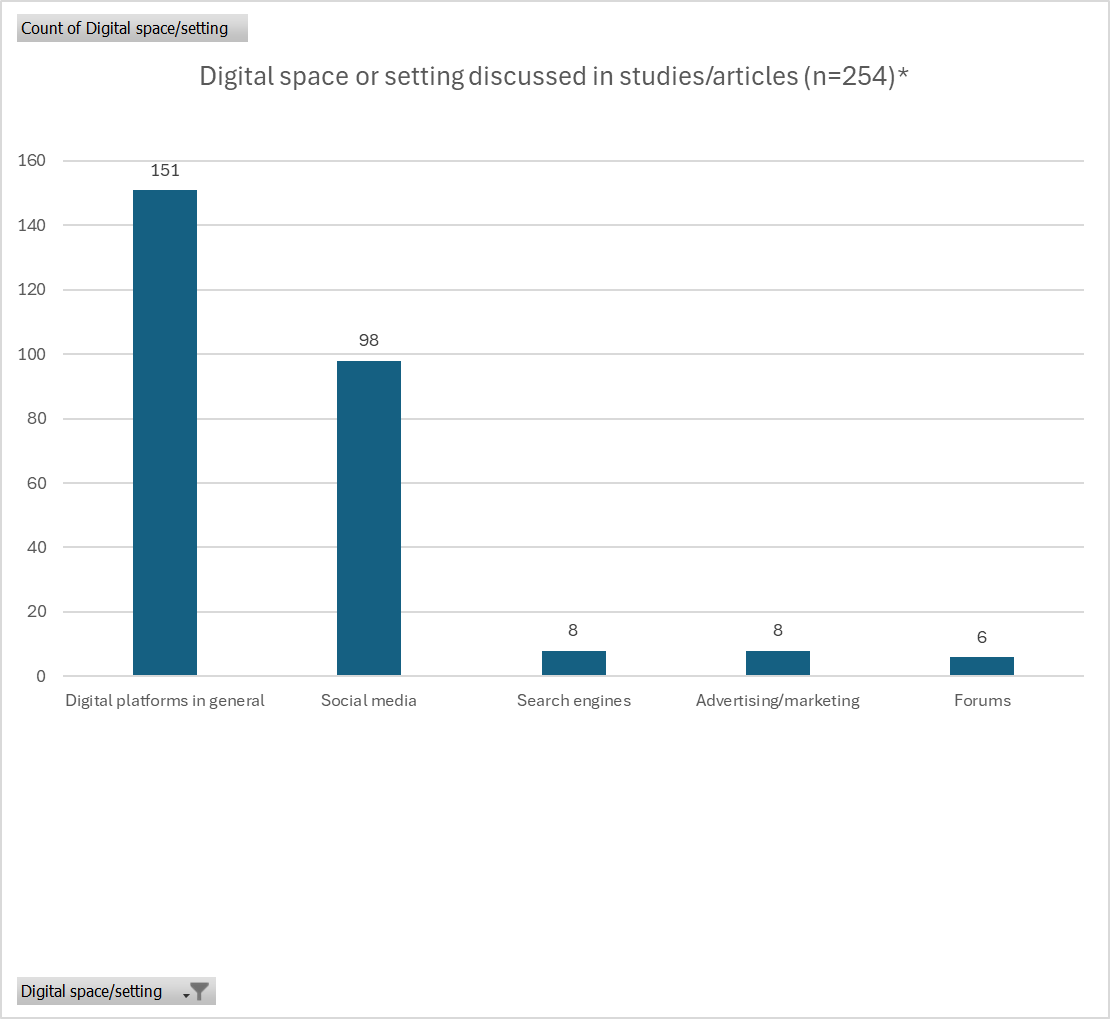
S7.**

**Figure S8.**


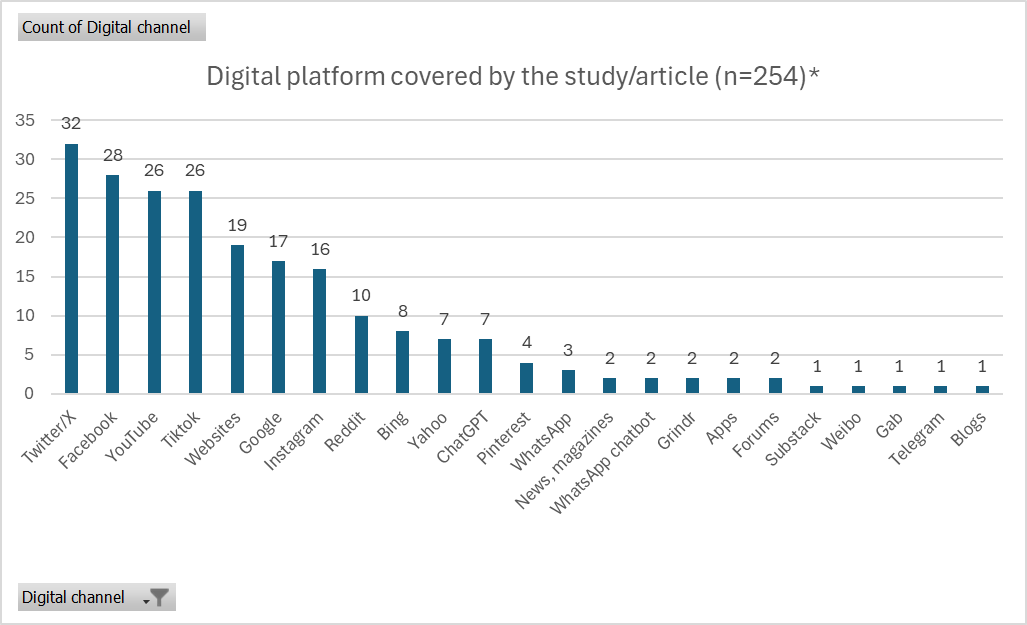


**Figure S9.**


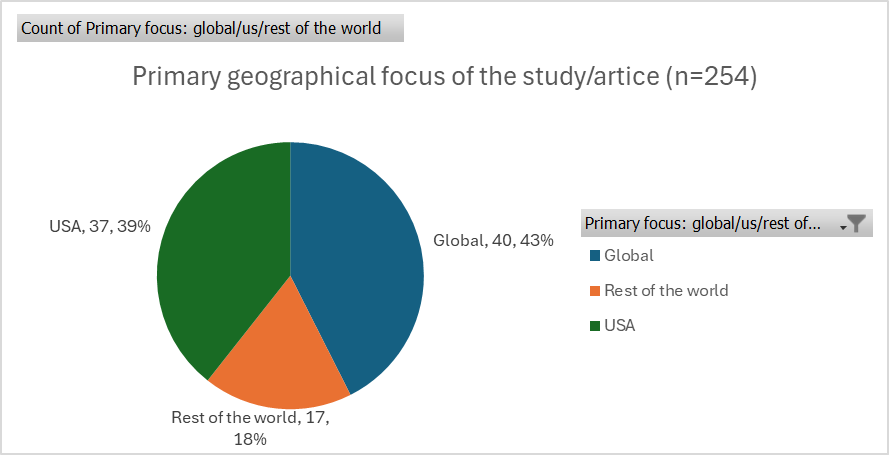


S10:


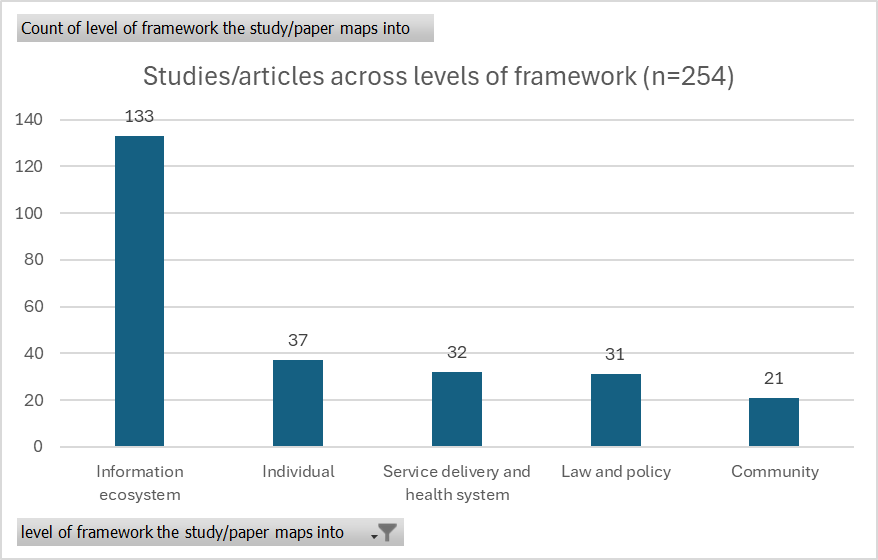


S11:


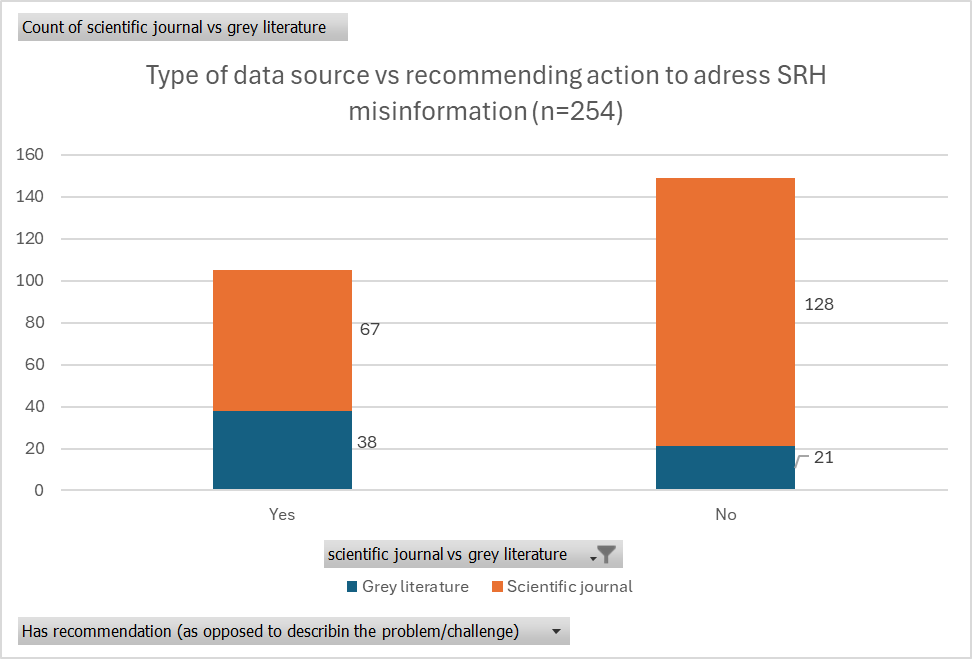


S12:

S13


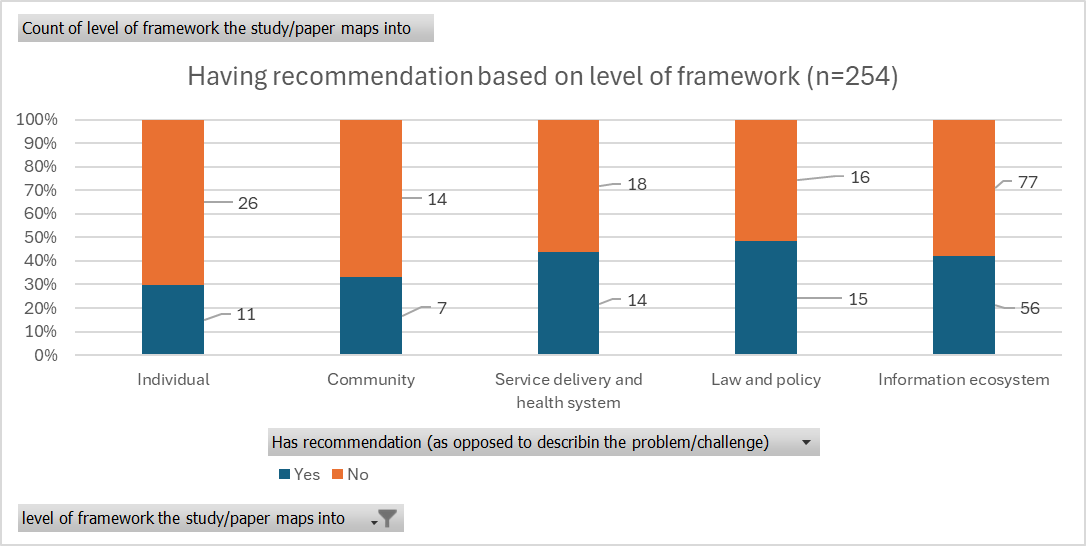


S14: Wordcloud of the terms used across the 254 studies, with underlying data

**Wordcloud generated by WordItOut web tool**


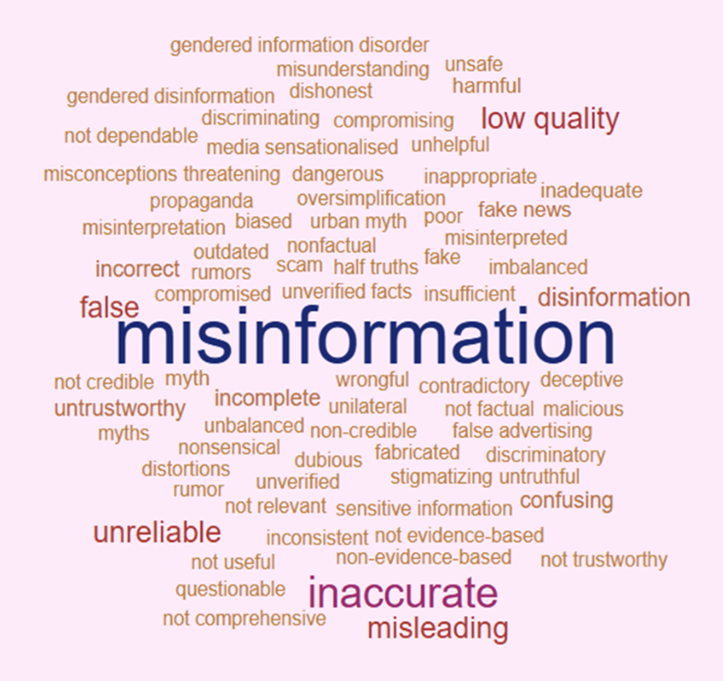


| **Words used to describe misinformation** | **Count of times used (one count per each of the 254 studies that used the term)** | **Proportion of use (%)** |
| --- | --- | --- |
| misinformation | 213 | 35.74% |
| inaccurate | 81 | 13.59% |
| low quality | 41 | 6.88% |
| misleading | 37 | 6.21% |
| unreliable | 37 | 6.21% |
| false | 35 | 5.87% |
| disinformation | 20 | 3.36% |
| untrustworthy | 11 | 1.85% |
| incorrect | 11 | 1.85% |
| incomplete | 10 | 1.68% |
| confusing | 9 | 1.51% |
| fake news | 7 | 1.17% |
| harmful | 5 | 0.84% |
| inadequate | 4 | 0.67% |
| myth | 4 | 0.67% |
| untruthful | 3 | 0.50% |
| poor | 2 | 0.34% |
| not comprehensive | 2 | 0.34% |
| stigmatizing | 2 | 0.34% |
| biased | 2 | 0.34% |
| unsafe | 2 | 0.34% |
| not credible | 2 | 0.34% |
| not factual | 2 | 0.34% |
| non-evidence-based | 2 | 0.34% |
| outdated | 2 | 0.34% |
| unverified facts | 1 | 0.17% |
| not useful | 1 | 0.17% |
| not evidence-based | 1 | 0.17% |
| misinterpretation | 1 | 0.17% |
| inappropriate | 1 | 0.17% |
| misinterpreted | 1 | 0.17% |
| gendered disinformation | 1 | 0.17% |
| dishonest | 1 | 0.17% |
| not trustworthy | 1 | 0.17% |
| misunderstanding | 1 | 0.17% |
| contradictory | 1 | 0.17% |
| compromising | 1 | 0.17% |
| wrongful | 1 | 0.17% |
| myths | 1 | 0.17% |
| gendered information disorder | 1 | 0.17% |
| non-credible | 1 | 0.17% |
| false advertising | 1 | 0.17% |
| dubious | 1 | 0.17% |
| media sensationalised | 1 | 0.17% |
| nonfactual | 1 | 0.17% |
| misconceptions | 1 | 0.17% |
| nonsensical | 1 | 0.17% |
| discriminatory | 1 | 0.17% |
| urban myth | 1 | 0.17% |
| dangerous | 1 | 0.17% |
| fabricated | 1 | 0.17% |
| unverified | 1 | 0.17% |
| fake | 1 | 0.17% |
| deceptive | 1 | 0.17% |
| propaganda | 1 | 0.17% |
| half truths | 1 | 0.17% |
| questionable | 1 | 0.17% |
| distortions of scientifically and medically accurate information | 1 | 0.17% |
| rumor | 1 | 0.17% |
| inconsistent | 1 | 0.17% |
| rumors | 1 | 0.17% |
| discriminating | 1 | 0.17% |
| scam | 1 | 0.17% |
| insufficient | 1 | 0.17% |
| compromised | 1 | 0.17% |
| malicious | 1 | 0.17% |
| threatening | 1 | 0.17% |
| not dependable | 1 | 0.17% |
| unbalanced | 1 | 0.17% |
| not relevant | 1 | 0.17% |
| unhelpful | 1 | 0.17% |
| sensitive information | 1 | 0.17% |
| unilateral | 1 | 0.17% |
| imbalanced | 1 | 0.17% |
| oversimplification | 1 | 0.17% |
| **Grand Total** | **596** | **100.00%** |
